# Dietary and Behavioral Strategies for Weight Loss and Weight Loss Maintenance: A Narrative Review

**DOI:** 10.3390/nu18010012

**Published:** 2025-12-19

**Authors:** Tomasz Żurawski, Anna Bartosiewicz

**Affiliations:** Department of Dietetics, Faculty of Health Sciences and Psychology, Collegium Medicum, University of Rzeszów, 35-959 Rzeszów, Poland; abartosiewicz@ur.edu.pl

**Keywords:** obesity, weight loss, weight loss maintenance

## Abstract

**Background**: Obesity is a multifactorial chronic disease associated with increased risk of metabolic disorders, reduced quality of life, and rising healthcare costs. Although weight reduction is achievable through various dietary approaches, maintaining the achieved results remains a major clinical challenge. This review aims to identify and discuss dietary, behavioral, and lifestyle strategies that support long-term weight loss and weight maintenance. **Methods**: A narrative literature review was conducted in two stages using PubMed, Scopus, and Google Scholar. The search included peer-reviewed studies published between 2015 and 2025 focusing on individuals with overweight and obesity. Randomized controlled trials, observational studies, systematic and narrative reviews, and meta-analyses discussing short-term and long-term weight loss outcomes and factors influencing the maintenance of reduced body weight were included. **Results**: Multiple nutritional strategies, including continuous and intermittent energy restriction, very low-calorie diets, and macronutrient modification, can be effective for weight reduction. However, long-term outcomes are primarily dependent on adherence and consistent implementation of recommendations. Behavioral and psychological factors, such as emotional eating, dietary fatigue, and unrealistic expectations, often contribute to weight regain. Social support, personalized dietary strategies, and sustainable lifestyle habits are key determinants of maintaining weight loss. The current evidence base is limited by the scarcity of long-term follow-up studies and high methodological variability across interventions. **Conclusions**: Long-term weight management requires a comprehensive, individualized approach that integrates dietary strategies with behavioral and lifestyle support. Strengthening adherence and addressing psychological and environmental factors may significantly improve the effectiveness and durability of obesity treatment.

## 1. Introduction

Obesity is a complex, multifactorial chronic disease characterized by excessive accumulation of adipose tissue [1]. Its development is the result of a complex interaction of genetic, environmental, socio-economic, and lifestyle factors, including diet and level of physical activity [2,3]. According to data from the World Health Organization (WHO), the number of people affected by obesity continues to increase globally, which makes obesity one of the top public health challenges [4]. It is currently estimated that about 650 million adults and 340 million children and adolescents aged 5–19 years suffer from obesity [5]. Moreover, forecasts from the World Obesity Federation (WOF) indicate that by 2035 the percentage of individuals with overweight and obesity may increase to 51% of the global population, resulting in significant economic burdens and major challenges for healthcare systems [6].

The consequences of obesity are multidimensional and include the risk of developing numerous chronic diseases, such as type 2 diabetes, cardiovascular diseases, and certain cancers, as well as a significant impact on patients’ quality of life and lifespan [1,7]. Obesity is also associated with serious emotional and social challenges, including stigma, low self-esteem, and mental health conditions [8]. The global obesity epidemic not only increases the health burden but also leads to substantial economic costs. It is estimated that expenditures related to overweight, and obesity could increase from $1.96 trillion in 2020 to $4.32 trillion in 2035, representing a rise in the share of global GDP from 2.4% to 2.9% [6].

Weight reduction is a key component in combating obesity. According to clinical guidelines, success in obesity treatment is most often defined as a reduction in body weight by at least 5–15% [9]. Such changes are associated not only with improved metabolic health but also with a reduction in the risk of comorbidities. However, success in obesity management is a multidimensional concept, and patients’ subjective goals such as improved appearance or better mental wellbeing often go beyond medically established definitions of success. Therefore, it is important to take these individual goals into account, as they play a critical role in defining success and highlight the complexity and multifaceted nature of effective obesity treatment [10].

Achieving and maintaining long-term weight loss is a difficult challenge. Data from earlier long-term analyses indicate that within two years of completing a weight loss program, over 50% of individuals regain their lost weight, and within five years, this percentage exceeds 80% [11]. This process is driven by several biological adaptations, such as a decreased metabolic rate and changes in hunger-regulating hormones, including increased ghrelin and decreased leptin. At the same time, a return to previous eating habits and insufficient adherence to dietary recommendations make maintaining the achieved results even more difficult. Environmental factors, such as the easy availability of high calorie foods and limited social support, further contribute to weight regain [12].

Pharmacological treatments for obesity, including the rapidly increasing use of GLP-1 receptor agonists, have expanded contemporary therapeutic options [13], yet they do not replace the need for durable lifestyle change. Evidence indicates that weight regain frequently occurs after discontinuation of anti-obesity medications [14], which highlights the importance of sustained nutritional and behavioral strategies alongside any pharmacological approach. Medically assisted weight loss may also be accompanied by a disproportionate reduction in fat free mass [15], further emphasizing the relevance of lifestyle components in supporting metabolic health during treatment. These considerations frame nonpharmacological strategies as a central element of long-term obesity management, both for individuals using pharmacotherapy and for those relying solely on lifestyle-based approaches. For this reason, the present review deliberately does not cover bariatric surgery or pharmacological treatment of obesity as primary interventions, focusing instead on dietary, behavioral, and lifestyle strategies that form the foundation for long-term weight loss maintenance in routine clinical practice.

This review aims to identify and discuss effective strategies that support obesity management by synthesizing evidence on dietary approaches together with key physiological, behavioral and psychosocial determinants of weight related outcomes. In contrast to earlier reviews that focused mainly on the effects of specific dietary patterns with limited consideration of these broader factors [16], the present work integrates evidence on appetite regulation, sleep and physical activity patterns, adherence related barriers and psychosocial influences that shape both weight loss and its longer term trajectory. It also incorporates data describing individuals who have successfully maintained weight loss, providing insight into behavioral consistency, lifestyle structure and self-regulation strategies associated with durable results. This approach establishes a comprehensive context for understanding the factors that support both the initiation and the continuation of effective weight management and may inform clinical practice for individuals managing excess body weight with or without pharmacological or surgical treatment.

## 2. Materials and Methods

The literature review was conducted in two stages using three databases: PubMed, Scopus, and Google Scholar. The aim was to obtain current, reliable, and comprehensive data on long-term weight loss in individuals with overweight and obesity, to identify potential strategies to support the maintenance of weight loss, and to analyze factors determining the durability of changes and adherence to dietary recommendations both during and after the reduction process.

In the first stage, a broad search was carried out using the main keywords such as obesity, long-term weight loss, sustainable weight loss, prolonged weight loss, lasting weight loss, persistent weight loss, weight maintenance, weight management, weight regain, fat loss, body fat reduction, permanent weight loss, prevention weight regain, management of obesity and obesity management. The phrases were combined using the Boolean operators “AND” and “OR”, which enabled the inclusion of both a wide range of related thematic issues and a variety of methodological approaches. Additionally, the Boolean operator “NOT” was used to exclude publications on bariatric surgery and obesity pharmacotherapy.

Google Scholar was used as a supplementary source, enabling access to publications that might not have been indexed in PubMed and Scopus. Due to the limited functionality and transparency of this database, all found items were subjected to manual selection for compliance with the adopted methodological criteria.

The second stage of the search aimed to identify literature focusing on more specific aspects of long-term weight loss. The search was based on phrases such as rapid weight loss, fast weight loss, rate of weight loss, weight loss of pace, energy restriction, very low-calorie diet, low-calorie diet, weight regain, diet patterns, low-carb diet, low-fat diet, high-protein diet, different macronutrient diets, personalized nutrition, individual diet, tailored nutrition, nutrition intervention, satiety, hunger control, appetite regulation, foods and satiety, dietary strategies, sleep weight loss, sleep quality, sleep duration, lifestyle changes, weight maintenance, long-term habits, behavioral habits, behavioral interventions, psychological strategies, self-monitoring, adherence, physical activity, exercise, resistance training. All the terms mentioned were combined with phrases such as obesity or weight loss to limit the search to studies on individuals with overweight and obesity. As in Stage I, the operator “NOT” was used to exclude studies related to pharmacotherapy and surgical treatment of obesity.

### 2.1. Inclusion and Exclusion Criteria

The inclusion and exclusion criteria were the same in both stages. Only peer-reviewed scientific publications published in English between 2015 and 2025 were included. This restriction of the literature search to the period 2015–2025 was applied to ensure coherence and clarity of the narrative synthesis given the broad thematic scope of the review. An unrestricted time frame in this type of work could increase the risk of selective study inclusion and inconsistency in the interpretation of findings. Randomized controlled trials (RCTs), systematic and narrative reviews, metaanalyses, and observational studies that included adults with overweight and obesity, analyzed the effects of short-term and long-term weight loss, assessed strategies to support weight maintenance after weight loss, and addressed the consequences and effectiveness of adherence to dietary and lifestyle recommendations were eligible for analysis. Publications on pharmacological and surgical treatment, studies conducted on animal models, studies without original scientific value (e.g., editorial comments, letters to the editor, expert opinions), and articles published in languages other than English were excluded.

### 2.2. Selection Process

In the first stage of the search, 304 publications were identified from the PubMed database, of which 150 were qualified for further analysis after analysis of titles and abstracts. 169 publications were obtained from the Scopus database, of which 42 were retained after the selection and removal of duplicates. 14 publications meeting the criteria were obtained from Google Scholar. In total, 206 works from the first stage were qualified for further full-text analysis.

In the second stage of the search, 1105 publications were identified. After removing duplicates and assessing titles and abstracts according to the adopted criteria, 310 works were qualified for further analysis.

Thus, the total number of publications subjected to full-text analysis was 516. As a result of a detailed methodological and substantive assessment, 110 publications were included in the main review. Additionally, due to the narrative nature of the study, 34 works identified during the citation review and selective manual search were included. In total, 144 publications were included in the review, which constitute the basis for the presented analysis and conclusions.

A narrative review approach was selected due to the complexity of obesity as a chronic disease, which involves the simultaneous influence of biological, nutritional, behavioral and environmental factors. The available studies addressing these areas differ substantially in study design, definitions of interventions, protocol duration, participant characteristics, and outcome measures used to assess effectiveness. Such broad heterogeneity prevents the development of uniform criteria required for conducting a systematic review without excluding clinically relevant evidence. A narrative format allows integration of findings from diverse types of studies and presents them in a manner that reflects the multifactorial nature of obesity treatment.

This approach has inherent methodological limitations. It does not allow for a formal assessment of the strength of evidence or for quantification of heterogeneity, and the synthesis is interpretative in nature. The risk of selection bias was reduced by applying transparent search and selection criteria, although narrative synthesis cannot replace the structured assessment offered by systematic review methods. In this work, the narrative design enabled the inclusion and joint interpretation of the various dimensions of obesity treatment within a single comprehensive framework.

Due to the substantial diversity of study types and designs, a single standardized tool for assessing methodological quality or risk of bias could not be applied. A formal risk of bias assessment was therefore not performed. Study selection was based on clearly de-fined search and inclusion criteria to reduce the risk of selection bias.

**Figure 1:**
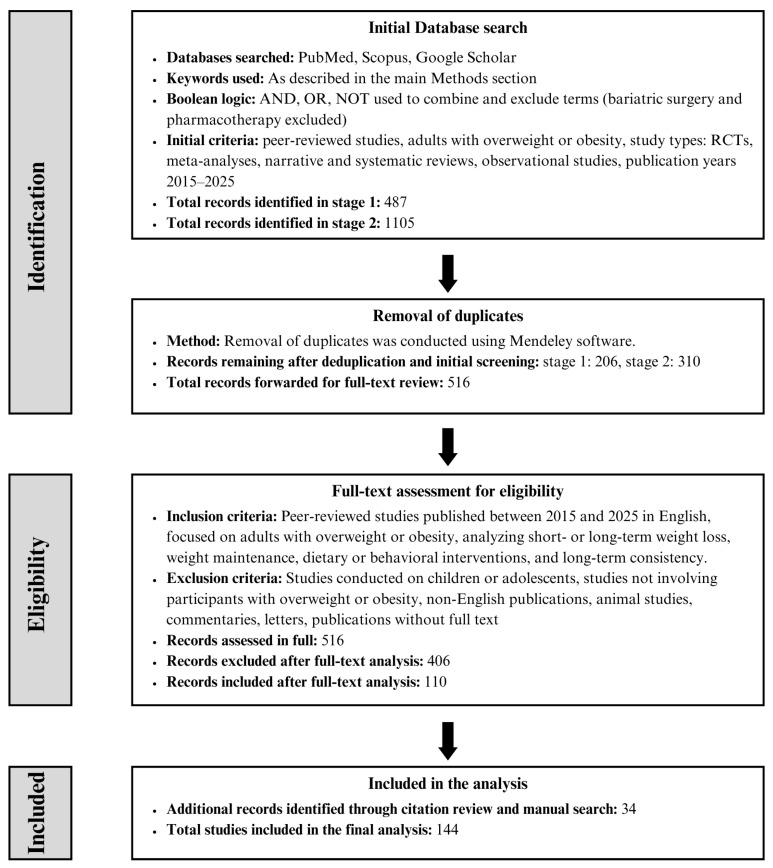
Flow chart of the search and selection of publications for the narrative review.

The exact literature selection process, along with the number of papers identified at each stage and duplicates removed, is presented graphically in Figure 1.

## 3. Results

### 3.1. Nutritional Interventions in Obesity

Fat tissue reduction is only possible under negative energy balance, when the energy expended exceeds, the energy supplied from food. In such circumstances, the body is forced to draw on stored energy reserves, primarily in the form of fat tissue, to meet its metabolic needs [17]. The key factor in successful weight loss is the introduction of a controlled calorie deficit, in line with the basic principles of thermodynamics. Although this process may seem simple in theory, in practice it requires precise planning and long-term commitment. Various factors, including metabolic adaptations, level of physical activity and hormonal changes, can influence both the rate and effectiveness of weight loss. For this reason, the process should be approached in a balanced manner and based on scientific recommendations.

#### 3.1.1. Energy Restriction Strategies

##### Very Low Calorie Diets

Very low calorie diets (VLCD/VLED) and very low calorie ketogenic diets (VLCKD) are primarily used in patients with severe obesity or metabolic complications, especially in situations that require rapid and substantial weight loss. VLCD/VLED protocols provide fewer than 800 kcal per day, while VLCKD additionally limits carbohydrate intake to 30 to 50 g per day, inducing a state of ketosis. After the intensive reduction phase, there is a reintroduction period during which energy intake is increased to help maintain the results [18,19]. Both strategies are often based on commercially prepared dietary products, which facilitate adherence and reduce the risk of nutritional errors [18,20]. Due to the severe caloric restriction, these interventions require strict medical supervision and supplementation of essential vitamins and minerals. They are used in contexts such as preparation for bariatric surgery, treatment of morbid obesity, and improvement of metabolic parameters [18].

Research on very low calorie diets consistently demonstrates substantial short-term reductions in body weight. Across interventions lasting from several to twelve weeks, weight loss typically ranges from approximately 10 to 17 kg [18,21], with higher energy deficits associated with faster reductions. In protocols providing about 400 kcal per day, reported losses ranged from approximately 5 kg after two weeks to more than 25 kg after several months, whereas interventions providing about 800 kcal produced smaller but directionally comparable effects [19]. The available studies differ markedly in terms of prescribed energy intake, intervention duration and inclusion criteria, which limits the direct comparability of their outcomes. Some analyses also incorporate overlapping datasets, which may overestimate the precision of effect estimates [18,21]. Despite this heterogeneity, the overall pattern of findings consistently indicates rapid and substantial short-term weight loss.

Data on the long-term effectiveness of very low calorie diets remain limited. Most available publications focus on short-term outcomes, and studies with follow-up periods exceeding 12 months are scarce. In one analysis, the average weight loss after more than one year was 21.48 kg [18], although this estimate was based on only two included studies. Broader evidence comes from research on VLED protocols supported by behavioral interventions. After 12 months, average weight loss was 10.3 kg, compared to 6.4 kg in groups receiving behavioral programs alone. After 24 months these values decreased to 4.2 and 2.8 kg, and after 38 to 60 months to 3.4 and 2.1 kg respectively [22]. These findings indicate a gradual attenuation of initial effects over time and highlight the role of behavioral support in limiting the regain of body weight.

Very low calorie diets also lead to meaningful short-term improvements in metabolic parameters. The most commonly reported changes include reductions in fasting glucose, markers of insulin resistance, HbA1c, triglycerides, total cholesterol, blood pressure, and inflammatory markers [18,22]. The magnitude of these effects varies across studies due to differences in intervention protocols and participant characteristics. Available data refer predominantly to the active weight loss phase, and the long-term durability of these metabolic improvements remains uncertain because most studies do not include extended follow-up.

Very low calorie interventions may be accompanied by adverse effects, particularly in the initial phase of the protocol. The most commonly reported symptoms include fatigue, headaches, dizziness, constipation, and feeling cold, which are attributed to the substantial energy deficit and limited carbohydrate intake [18,22]. Some analyses have also documented changes in gut microbiota composition, including reductions in microbial diversity, potentially related to low fiber intake and the use of processed meal replacements [20]. Available evidence refers mainly to the active weight loss period, and the long-term significance of these observations remains uncertain.

Sex related differences have been reported in studies assessing the effects of very low calorie diets, although the magnitude and consistency of these findings are limited. In one study men experienced greater and more rapid short-term weight loss, whereas women showed more stable outcomes in the medium term [23]. Other publications have noted distinct changes in body composition and metabolic response, including greater losses of lean mass and larger reductions in HDL cholesterol in women and greater reductions in fat mass and more pronounced improvements in metabolic syndrome indicators in men [24]. In postmenopausal women a decrease in hip bone mineral density was observed during intensive weight loss [25]. Long-term data remain scarce and variation in study designs and participant characteristics prevents firm conclusions regarding whether the reported sex related differences occur consistently or represent durable patterns.

##### Intermittent Energy Restriction (IER)

Intermittent energy restriction (IER) refers to dietary strategies in which calorie intake is limited to specific time periods or designated days of the week. The most commonly examined models include periodic fasting (PF), which involves complete or near complete fasting for one or several days per week, typically on an irregular schedule depending on study protocol, and alternate day fasting (ADF), in which fasting days with drastically reduced or no energy intake alternate with days of regular eating. Another frequently studied form of IER is time-restricted eating (TRE), where food consumption is limited to a specific daily window of 6 to 12 h, with the remaining hours allocated to fasting [26].

The effectiveness of IER in reducing body weight has been compared with both continuous energy restriction (CR) and ad libitum dietary approaches. Available metaanalyses include studies with diverse designs, where IER was evaluated against one or both strategies, which substantially limits the direct comparability of results. Additional sources of variability include differences in intervention duration and participant characteristics. Despite the growing number of publications, long-term data remain scarce, and the current evidence base does not allow for a definitive assessment of the durability or safety of IER. A summary of findings on body weight outcomes is presented in Table 1.

The short-term efficacy of IER for reducing body weight remains inconsistent and is strongly infuenced by the characteristics of the control group and the methodological design of the studies. In analyses comparing IER with ad libitum or mixed interventions (ad libitum combined with CR) [26,27,30,32], slightly greater weight loss was observed in the IER groups, although the differences were small. In studies comparing IER with CR [28,31], no advantage of either strategy was demonstrated. An exception is one study in which ADF led to greater weight loss (1.66 kg) compared to CR in less than 3 months [29]. However, this finding was derived from a single protocol.

Data on the long-term effectiveness of IER are limited, and most studies report follow-up periods shorter than 12 months. In analyses lasting 6 to 8 months, IER showed a slight advantage over ad libitum diets, although this difference disappeared in interventions lasting 10 to 14 months [26]. Most studies comparing IER with CR do not demonstrate significant differences in effectiveness beyond 6 months [28,29,31]. Several publications report weight plateau or partial weight regain in both types of interventions [31]. Overall, the available evidence indicates that long-term outcomes of IER do not differ substantially from those observed with CR, although interpretation remains constrained by the small number of studies with extended follow-up.

Data comparing different IER models, indicate that the relative effectiveness of these protocols for weight reduction remains inconsistent. Several analyses report similar outcomes across various IER approaches [26]. Some publications suggest that more restrictive strategies, such as ADF, may lead to greater weight loss compared to TRE [30], although these findings are not consistent. In TRE protocols, shorter eating windows of 6 to 8 h were associated with an average weight loss of 2.73 kg, whereas longer windows of 10 to 12 h did not produce significant changes [27]. In one study involving individuals with overweight and obesity, only PF protocols achieved weight loss exceeding 5% [33]. Interpretation of these findings is constrained by the limited number of studies directly comparing different IER models, which restricts the ability to draw firm conclusions regarding their relative effectiveness.

Metaanalyses have reported beneficial changes in insulin sensitivity, fasting glucose, triglycerides, and LDL cholesterol, particularly in individuals with insulin resistance or metabolic syndrome [30,33]. These findings are derived from studies with heterogeneous populations and intervention protocols, which limits direct comparability and the ability to attribute observed effects specifically to IER. Studies comparing IER with CR have not demonstrated significant differences in metabolic outcomes between these approaches [31,32].

##### Continuous Calories Restriction (CR)

CR is the most widely implemented and clinically recommended strategy for the treatment of obesity [34]. In clinical studies, CR typically involves maintaining a constant daily energy deficit of 500 to 750 kcal, which corresponds to energy intakes of approximately 1200 to 1500 kcal per day for women and 1500 to 1800 kcal per day for men [35]. These protocols lead to gradual weight loss, and their effectiveness results from sustaining a stable negative energy balance [34].

Short term interventions based on continuous caloric restriction produce clinically meaningful reductions in body weight. In a 6-month study, a 25% reduction in energy intake resulted in a weight loss of 7.06% in one dietary arm and 6.21% in the control group [36]. A meta-analysis in postmenopausal women reported a mean weight loss of 6.55 kg across interventions lasting 3 to 12 months, with most studies spanning 3 to 4 months [37]. Another metaanalysis indicated that most CR interventions lasting at least 3 months achieve reductions exceeding 5% of baseline body weight [29].

Assessing the long-term effectiveness of CR is challenging due to the limited duration of most available studies. Most interventions do not exceed 12 months [38,39,40,41,42], and the dietary phase commonly lasts for a shorter period followed by a follow up. In some studies, the weight loss achieved during the intervention was maintained [38], while others reported gradual weight regain [40,41,42]. In a 24 months study, a reduction of 6 kg achieved during the first 3 months was maintained until month 12, after which partial regain occurred, resulting in a final reduction of 4 kg at 24 months [41].

#### 3.1.2. The Role of Diet Composition in Weight Loss

In addition to energy restriction strategies, studies on weight reduction also examine the role of diet composition. Existing evidence indicates that the magnitude of the energy deficit remains the primary factor associated with changes in body weight [17]. At the same time, numerous interventions evaluate how macronutrient proportions and the selection of specific food types relate to weight loss outcomes and selected metabolic parameters.

##### Low Carb (LC) and Low Fat (LF) Diets

LC and LF diets are among the most frequently compared macronutrient modification interventions in obesity treatment studies.

Available data indicate that LC and LF produce comparable short-term reductions in body weight. In 6 month interventions, mean weight loss ranged from 4 to 5 kg, with no statistically significant differences between the diets [43]. The definitions applied to both dietary approaches varied substantially. LC interventions included models providing approximately 20% of energy from carbohydrates as well as variants delivering 35 to 40%. LF diets were defined as providing less than 30% of energy from fat, which encompassed both very low fat and moderately restricted fat intake [43].

In studies applying more stringent criteria for LC, such as carbohydrate intake below 20% of energy or 20 to 40 g/day during the initial phase, LC was associated with greater weight loss than LF. The mean difference was 2.17 kg after at least 6 months of intervention [44]. Across the included studies, LC was generally implemented ad libitum, whereas LF interventions assumed energy restriction. Reported lipid changes in LC groups included lower triglyceride concentrations and higher HDL cholesterol and LDL cholesterol levels [44].

In a 12-months intervention without a centrally imposed energy deficit, LC and LF produced comparable reductions in body weight (6.0 kg and 5.3 kg respectively), accompanied by substantial individual variability, ranging from weight losses of up to 25 kg to weight gain in some participants [45]. Both groups showed reductions in fasting glucose, insulin, and triglycerides, with no statistically significant differences between interventions. LC was also associated with increased HDL cholesterol and a modest increase in LDL cholesterol [45]. Secondary analyses indicated that in some studies, greater reductions in body weight co-occurred with qualitative changes in dietary patterns, including decreased intake of highly processed foods and increased consumption of nutrient-dense products. These changes were associated with an unintentional reduction in energy intake of approximately 500 to 600 kcal/day [46].

In a controlled metabolic study conducted under isocaloric and tightly regulated dietary conditions, LC was associated with higher fat oxidation and lower insulin concentrations, whereas LF resulted in greater adipose tissue loss. Differences in total body mass loss were minimal [47].

##### Ketogenic Diet (KD)

KD characterized by a very low carbohydrate intake below 50 g/day and a high fat content typically providing 70 to 80% of total energy, results in the maintenance of state of ketosis [48].

Among women with overweight and polycystic ovary syndrome (PCOS), a mean reduction in total body weight of 9.13 kg was reported over a period of 4 to 24 weeks [49]. In another 12-weeks intervention, the mean reduction in total body weight was 18 kg in men and 11 kg in women with a similar relative reduction. The intervention included an initial energy restriction of 1200–1500 kcal/day, followed by continuation of KD without quantitative limits while maintaining its characteristic macronutrient distribution. The state of ketosis was maintained throughout the intervention. No control group was included in this study [50]. In a short term, tightly controlled metabolic study comparing isocaloric diets, KD was associated with higher fat oxidation and lower insulin concentrations, whereas the high carbohydrate diet resulted in greater adipose tissue loss. Differences in total body weight loss were, however, small [48].

Across studies investigating KD, characteristic changes in lipid parameters were reported, including reductions in triglyceride concentrations and increases in HDL cholesterol. In some interventions, increases in LDL cholesterol were also observed.

In comparisons conducted under matched energy intake, KD did not demonstrate superiority over higher carbohydrate diets in reducing body weight [48]. Regarding appetite and satiety, studies reported reductions in appetite during KD as well as no measurable effect in other interventions, resulting in inconsistent findings [51].

Long-term data on the effectiveness of KD and the durability of associated metabolic changes remain limited.

##### Mediterranean Diet (MedDiet)

MedDiet, characterized by a high intake of monounsaturated fatty acids, fiber, and antioxidant compounds, is one of the most studied dietary models in the context of cardiovascular disease and type 2 diabetes prevention [52].

In a 16-weeks intervention among postmenopausal women with overweight and type 2 diabetes, a mean weight loss of 7.6 kg was observed during MedDiet, whereas a LC group the reduction amounted to 9.1 kg [53]. In interventions lasting at least 12 months, weight loss during MedDiet ranged from 3.8 to 10.1 kg. These studies reported greater reductions in body weight compared with LF interventions and values comparable to those observed during LC diets [54].

In individuals with excess body weight and comorbid type 2 diabetes, MedDiet was associated with reductions in HbA1c and fasting glucose [52]. Among individuals without diabetes, these changes were comparable to those observed in other dietary interventions [54]. Reductions in blood pressure were also reported during MedDiet. In participants with type 2 diabetes, greater decreases in blood pressure were observed compared with LF [52], whereas in long term studies no significant differences in blood pressure were found between MedDiet and other dietary strategies [54]. For lipid parameters, findings during MedDiet were generally similar to those of other dietary interventions, except for triglycerides, which showed greater reductions after at least 12 months of adherence to MedDiet [54].

##### High Protein Diets

High-protein diets represent a distinct approach within weight loss strategies. In typical dietary patterns, protein provides approximately 15% of total energy intake [43], whereas in high protein diets this proportion may reach 30% or more [55,56]. Across studies, definitions of high protein diets varied and included both an increased percentage of energy derived from protein and relative values expressed in grams per kilogram of body weight per day.

Comparative studies reported greater reductions in body weight in groups following high protein diets than in those following standard diets under similar energy deficits [56]. Some studies also demonstrated greater decreases in BMI in groups adhering to high protein interventions [56]. In older adults with overweight and obesity, a diet providing 1.4 g of protein/kg/day resulted in a body weight reduction of approximately 3.3% and a smaller loss of lean body mass compared with a control diet providing 0.8 g/kg/day [57]. In a 6-months intervention among individuals with metabolic syndrome, a high protein diet providing 1.34 g/kg/day with an identical energy deficit of 500 kcal/day led to a greater weight loss (7.0 kg and 5.1 kg respectively) and a more pronounced decrease in waist circumference compared with a control diet providing 0.8 g/kg/day [58].

In populations with type 2 diabetes, findings regarding the effects of high protein diets on metabolic parameters were inconsistent. Some studies reported no significant changes in glycemic control or blood pressure compared with control diets, although reductions in LDL cholesterol, total cholesterol, triglycerides, and HOMA-IR were noted [59]. Other studies reported improvements in glycemic control along with reductions in HOMA-IR [55]. Among individuals with obesity but without metabolic comorbidities, no significant differences in lipid parameters were observed between high protein diets and control diets under comparable energy deficits [56].

### 3.2. Evidence on Mechanisms and Modifiable Factors Influencing Weight Loss

A growing body of research indicates that responses to weight loss interventions are shaped not only by the magnitude of the energy deficit but also by a range of physiological, psychological, behavioral and environmental processes. Published findings describe mechanisms that may influence the pace of weight reduction, the degree of metabolic and hormonal adaptation, and the capacity to maintain the behaviors required throughout treatment. The following subsections synthesize evidence on these processes, including metabolic adaptation, hunger and satiety regulation, psychological and behavioral determinants, and selected lifestyle factors such as sleep and physical activity.

#### 3.2.1. Metabolic Adaptation

Metabolic adaptation, defined as a reduction in total energy expenditure that exceeds the decrease predicted from the loss of fat and fat free mass, has been proposed as one of the factors that may hinder long-term weight loss maintenance [60]. However, findings across studies are inconsistent.

In one study, 9 weeks of weight loss resulted in a reduction in total energy expenditure of approximately 100 kcal/day. After 4 weeks of weight stabilization, the magnitude of this change decreased by half and was no longer detectable after 1 year [61]. In another study in which participants achieved a body weight reduction of approximately 16%, mean metabolic adaptation reached 46 kcal/day. The magnitude of this adaptation was a significant predictor of the time required to reach the targeted weight loss, with greater adaptation being associated with a slower rate of progress [62]. A systematic review reported that the extent of metabolic adaptation varies and depends, among other factors, on the degree of energy deficit and participants body composition [60]. Across available studies, these adaptations were most commonly associated with the current energy balance.

The most pronounced metabolic adaptations were observed among participants of The Biggest Loser program. At the end of the 30-weeks intervention, mean metabolic adaptation reached approximately 275 kcal/day, and during the 6-year follow-up it was approximately 500 kcal/day, despite substantial weight regain in most participants. The magnitude of metabolic adaptation did not show a clear association with the amount of weight regained, suggesting that reductions in energy expenditure may occur independently of long-term weight changes in some individuals [63]. Several studies also examined the relationship between physical activity levels and the extent of metabolic adaptation. In some investigations, higher physical activity was associated with greater reductions in total energy expenditure during weight loss, although this association was not consistently observed across all studies [60].

In a study conducted in individuals with obesity, planned interruptions of energy restriction with periods of energy balance were associated with a smaller metabolic adaptation compared with continuous restriction. The reduction in body composition adjusted resting energy expenditure was approximately 85 kcal per day in the intermittent protocol and approximately 180 kcal per day during continuous restriction, despite an identical total time spent in energy deficit. The overall duration of the intervention was longer due to the inclusion of interruption periods [64].

#### 3.2.2. Hunger and Satiety Regulation and Modifiable Contributors to Appetite Control

Weight loss is associated with neurohormonal changes that may contribute to increased appetite. A mathematical model of energy balance in a study of individuals with overweight and obesity showed that appetite increased by approximately 100 kcal/day for each kg of body weight lost [65]. These changes occurred alongside alterations in hormones involved in the regulation of hunger and satiety.

In the studies reviewed, increases in ghrelin concentrations were observed following weight loss. In individuals with obesity who lost approximately 13% of their body weight, total ghrelin levels increased by 23%, and remained 16% above baseline after 12 months of weight maintenance [66]. Another study reported an 18 percent increase in ghrelin after 6 months of weight loss, which remained elevated at the 24 month follow up despite partial weight regain [67].

Weight loss was also associated with changes in satiety related hormones. Reductions in peptide YY, GLP 1, cholecystokinin, and amylin concentrations were reported after weight loss [68]. Compared with lean individuals, those with obesity demonstrated lower postprandial responses of peptide YY and GLP 1 both before and after weight reduction [45]. In some observations, reduced concentrations of these hormones persisted for several months following the end of the intervention [68].

Leptin represents another component of long-term body weight regulation. Individuals with obesity have been reported to exhibit high circulating leptin concentrations [69,70]. Although weight loss is associated with a marked decrease in leptin concentration [70]. In one study, a greater early decrease in leptin during a dietary intervention was associated with greater weight loss at 12 months [71].

Differences were also observed in central nervous system responses to food related cues. Functional MRI studies showed that individuals with obesity exhibited greater activation of reward related brain regions in response to food stimuli [72]. One study found higher postprandial activation of the prefrontal cortex in individuals with class III obesity compared with those with class I or II obesity [73]. Another study reported that activation of reward related regions persisted even after the onset of satiety [74].

Although most available data derive from short-term studies, current findings suggest that approaches reducing hunger may support weight loss and adherence to dietary recommendations [75].

In studies evaluating dietary composition, higher protein intake was associated with reduced hunger and greater satiety. These effects coincided with changes in GLP-1, PYY, and ghrelin concentrations as well as increased thermogenesis [76,77]. The literature also describes the protein leverage hypothesis, which proposes that insufficient protein intake may lead to compensatory increases in energy consumption from other macronutrients, although this concept requires further verification [78]. In several studies, increasing daily protein intake to 1.2–1.6 g/kg of body weight was associated with spontaneous reductions in energy intake under ad libitum conditions [76,77,79].

Increasing the consumption of low energy density foods such as vegetables, fruits, lean protein sources, whole grains, and low-fat dairy products was associated with larger meal volume at lower caloric cost [80]. Proposed mechanisms included gastric distension, mechanoreceptor stimulation, prolonged mastication, and increased secretion of satiety hormones such as GLP1 and PYY, alongside reductions in ghrelin concentrations [80,81].

A daily fiber intake of approximately 30 g was associated with improved appetite control, reduced spontaneous energy intake, and beneficial changes in gut microbiota [82,83]. Viscous fiber such as beta glucan and guar gum were identified as particularly effective in delaying gastric emptying and stimulating the release of GLP-1 and PYY [82]. In some studies, the effectiveness of fiber based approaches appeared to vary according to individual gut microbiota profiles [84].

Sensory and structural characteristics of foods also influenced appetite regulation. Solid foods generally induced greater satiety than liquids of comparable energy content, which has been attributed to longer chewing time and enhanced sensory stimulation [85]. Slower eating speed was associated with lower energy intake and a more attenuated postprandial rise in ghrelin [86]. Additionally, eating in distraction free environments without television or mobile devices supported better recognition of satiety cues and reduce the likelihood of overeating [87].

#### 3.2.3. Psychological, Behavioral and Social Determinants of Dietary Adherence

Psychological, behavioral and environmental factors play an important role in the effectiveness of interventions aimed at reducing body weight. Published data indicate that emotions, behavioral patterns and social conditions may influence adherence to dietary recommendations and the durability of achieved outcomes.

##### Emotions and Regulation of Eating Behavior

Emotional eating has been frequently observed among individuals with obesity and is associated with increased consumption of high calorie foods [88,89]. Difficulties in emotion regulation, including episodes of loss of control over eating and unplanned snacking, were also more commonly reported in this group [90]. Emotion suppression was linked to a higher risk of psychological difficulties, which may hinder the maintenance of modified behaviors [91].

In several publications, the use of emotion regulation techniques, such as mindfulness based practices, was associated with greater awareness of hunger cues and reduced impulsive eating [92]. In other reports, methods aimed at strengthening response control, including response inhibition training or modification of approach tendencies toward high calorie foods, were linked to improvements in impulse regulation [93].

Selected studies also indicated that body image perception may influence the stability of health related behaviors. Improvements in body acceptance were associated with greater durability of behaviors supporting weight control, particularly among individuals with low baseline satisfaction with their appearance [94].

##### Motivation, Habits and Cognitive Load

A gradual decline in motivation, diet related fatigue, and difficulty maintaining previously established habits were described as barriers to sustaining interventions [95,96]. Unrealistic expectations regarding the pace of weight loss were associated with a greater risk of frustration and discontinuation of efforts [97,98].

Several publications emphasized that rigid, restrictive dietary approaches and viewing weight loss as a temporary phase rather than a permanent lifestyle change were associated with a higher likelihood of returning to previous eating patterns [99,100].

Findings on self regulatory strategies indicated that regular monitoring of behaviors, planning, and flexible adjustment of actions were more frequently observed among individuals achieving long term results [94]. Habit based interventions led to modest reductions in body weight and greater consistency of behaviors supporting weight control [101]. Reports describing participant experiences suggested that elements reducing the number of daily food related decisions, such as a stable meal structure were perceived as easier to maintain [102].

##### Social and Environmental Factors

Weight related stigma was associated with higher stress levels and difficulties maintaining health promoting behaviors [103]. Negative beliefs about one’s body weight were linked to avoidance of behaviors supportive of weight management, including physical activity and greater risk of depressive symptoms [104]. Limited support from family or social networks was associated with a greater likelihood of discontinuing interventions [105].

Publications analyzing different forms of support indicated that group based approaches were associated with higher engagement and greater behavioral regularity [93,106]. In individual interventions the quality of the relationship between the practitioner and the participant was associated with better adherence [107]. Programs delivered exclusively online were more often characterized by reduced durability of outcomes, whereas hybrid models showed greater effectiveness [108,109].

##### Diet Personalization and Factors Related to Adherence

In several publications adherence was a stronger predictor of outcomes than the type of diet applied [110]. Higher adherence was observed when the dietary structure aligned with participants’ prior eating habits [110], while overly rigid dietary approaches were associated with lower long term sustainability [102]. In environments rich in highly palatable foods, eating in the absence of hunger was observed more frequently and was linked to activation of hedonic appetite regulation pathways [111]. Adjusting meal structure to participants’ preferences, including preferred meal frequency, was evaluated as easier to sustain [112]. The inclusion of ready-to-eat meal replacements was associated with slightly greater weight reduction and more regular adherence to the dietary plan [96]. Barriers to adherence also included the cost of food, product availability, and time required for meal preparation [113]. Findings from single studies suggested that personalization of dietary recommendations based on genetic profiles may support adherence, although data remain limited [114].

#### 3.2.4. Sleep and Its Influence on Appetite Regulation and Weight Loss

Available publications indicate that sleep may influence appetite regulation, food preferences, and the ability to maintain behaviors supporting weight reduction in individuals with excess body weight [115]. Guidelines from the American Academy of Sleep Medicine and the Sleep Research Society state that regularly sleeping fewer than 7 h per night is associated with a higher risk of weight gain and other adverse health outcomes [116].

In the studies analyzed, sleep restriction was associated with changes in the regulation of hunger and satiety hormones. Reductions in leptin levels and increases in ghrelin levels were reported and these changes were linked to greater appetite [117]. In some publications shorter sleep duration was associated with a greater preference for high calorie foods and increased impulsive eating. These phenomena were interpreted as reflecting increased reactivity of the reward system under conditions of insufficient sleep [118].

In one study involving individuals with excess body weight who slept fewer than 6.5 h per night extending sleep duration was associated with a reduction in daily energy intake despite the absence of additional dietary guidance [119]. In longer observations a favorable sleep profile that included adequate duration, regularity, quality, and efficiency correlated with greater reductions in body weight and fat mass during a 12 months weight loss program independently of the presence of obstructive sleep apnea [120]. A regular circadian rhythm and consistent sleep timing were associated with a lower risk of weight regain after the intervention [121].

Available publications also described that reductions in body weight may improve sleep parameters. Short term dietary interventions leading to weight loss were associated with improvements in sleep quality and reductions in the severity of obstructive sleep apnea [122]. Reductions in daytime sleepiness and improvements in daytime functioning were also observed regardless of the dietary macronutrient composition [123].

#### 3.2.5. Physical Activity as a Modifiable Factor Related to Weight Loss

Data from weight loss interventions indicate that physical activity alone usually leads to only modest reductions in body weight, and the response to exercise varies substantially between individuals [124]. More pronounced effects have been observed when physical activity was combined with dietary interventions or comprehensive lifestyle modification. In groups of individuals who maintained weight loss over the long term, regular engagement in physical activity was reported much more frequently. In one analysis, individuals with sustained results reported an average of 92 min of moderate or vigorous activity per day, with at least 60 min performed on approximately 73% of days [125].

The type of physical activity influenced the observed outcomes. Moderate-intensity aerobic exercise led to modest reductions in body weight, with the magnitude of the response depending on individual physiological differences [126]. High intensity interval training (HIIT) demonstrated comparable effects on reducing adipose tissue, particularly visceral fat, while requiring less time than traditional aerobic exercise [127].

Resistance training represented an important component of interventions aimed at weight reduction. Studies showed that resistance training helped limit the loss of lean body mass during energy restriction and may contribute to reductions in adipose tissue even when total body mass remained relatively stable [128]. The most favorable outcomes were observed in programs combining resistance training with aerobic exercise, which were associated with reductions in fat mass, preservation of muscle mass, and improvements in metabolic parameters [129].

Regular physical activity was also associated with improvements in selected health parameters independently of changes in body weight. Several publications reported beneficial effects on blood pressure, glycemic control, and lipid profile [130,131]. Physical activity has been linked to changes in appetite regulation, including alterations in leptin and ghrelin concentrations [131]. Some studies also highlighted positive effects of physical activity on mental health, including reductions in depressive symptoms, anxiety, and chronic stress [130].

### 3.3. Characteristics of Individuals Who Maintain Long-Term Weight Loss Outcomes

Available data from long-term studies and registries of individuals maintaining weight loss outcomes allow for the description of a set of behaviors that were more frequently observed in this population. They differ from studies focusing on the process of weight reduction itself, as they refer to the period after the intensive phase of weight loss, which enables the assessment of behavioral patterns associated with maintaining achieved results.

One of the most frequently reported elements was systematic monitoring of progress. Up to 78% of individuals maintaining weight loss outcomes weighed themselves at least once per week, and 44% did so daily [132]. Monitoring also included tracking energy intake and physical activity, which enabled early detection of deviations from established targets [12,132].

Regular physical activity was another consistently observed element. Individuals maintaining weight loss outcomes typically engaged in 200–300 min of moderate physical activity per week, often distributed across most days of the week [12]. The most frequently reported form of activity was walking, often supplemented with other forms of moderate-intensity movement [132]. The key factor was not the specific type of exercise but its regularity.

A stable structure of dietary habits was also more commonly present in this population. These individuals consumed meals at regular intervals, which supported appetite regulation and reduced the likelihood of impulsive eating [12,132]. Their diets were generally characterized by moderate energy density and a predominance of minimally processed foods [12,132,133]. Approximately 78% of individuals maintaining weight loss outcomes reported regular breakfast consumption [12,132]. Strategies aimed at reducing the risk of returning to previous behaviors were also reported, such as meal planning, preparing food in advance, using shopping lists, and limiting the availability of high calorie foods in the environment [132,133,134].

Reports describing the experiences of individuals maintaining weight loss outcomes also highlighted the role of changes in the way they thought about their health behaviors. These individuals more often perceived themselves as more active and health oriented, which could facilitate the integration of supportive behaviors into daily functioning [133]. Flexible responses to periodic lapses were another feature reported in this group. Episodes of increased energy intake or reduced physical activity led to a quick return to established routines, often through temporarily increasing activity or more attentive monitoring of intake [132,133].

An important factor supporting long-term maintenance of outcomes was social support. Reports more frequently noted support from family or close individuals, including shared meal preparation or joint physical activity [133]. Regular contact with a professional or participation in support groups was associated with greater consistency and maintenance of engagement [134].

## 4. Discussion

This review confirms that obesity is a multifactorial chronic disease, and that effective, sustained fat mass reduction requires an approach that extends beyond dietary strategies alone. Evidence indicates that weight management outcomes are shaped not only by energy restriction but also by behavioral factors, physical activity, sleep regulation, appetite related neurobiology, and environmental and psychosocial influences. While an energy deficit remains the fundamental driver of weight loss [17], various dietary, behavioral, and lifestyle interventions can be effective, although their results show considerable variability depending on the protocol used, the characteristics of the studied population, and the duration of the intervention. This heterogeneity highlights the need for cautious interpretation and for individualized clinical recommendations.

Very low calorie dietary interventions remain one of the few approaches capable of producing rapid and substantial reductions in body weight [18,19,21]. Available evidence also indicates that these short-term changes are accompanied by marked improvements in several metabolic parameters, including glycemia, lipid profile, blood pressure, and inflammatory markers [18,22]. Such effects may be particularly valuable in individuals at high metabolic risk, for whom rapid health improvement is a clinical priority. It should be emphasized, however, that most of these benefits have been reported in short-duration studies, whereas the number of investigations extending beyond 12 months remains limited [18,22], which constrains the ability to evaluate long-term durability and generalizability. These interventions are also associated with a specific risk profile. Studies indicate a greater loss of fat free mass in more restrictive and prolonged protocols, which may be relevant in individuals with low baseline muscle mass or sarcopenia. In postmenopausal women, reductions in bone mineral density have been observed, which may have implications for osteoporosis risk and justify closer monitoring of skeletal health [23,24,25]. Due to the very low energy intake and the simplified composition of commercial formula products, attention must also be given to the potential risk of deficiencies in selected vitamins and minerals [18,20,22]. Although the use of prepared dietary products facilitates precise energy control, it simultaneously limits opportunities to develop skills required for sustained dietary change. For this reason, specialist supervision and concurrent behavioral support are essential from the outset of the intervention rather than after its completion. In clinical practice, very low calorie dietary strategies may be valuable tools, but only in well defined situations, and should be implemented with caution, individualization, and full consideration of both their potential and their limitations.

The limitations of very low calorie diet protocols suggest that long-term obesity management requires a shift in emphasis from short-term, intensive interventions to strategies that are feasible in everyday settings and can be maintained outside of close clinical supervision. In this context, continuous CR and IER provide a practical framework for various dietary models to operate.

Comparative data indicate that, with a balanced energy intake, CR and IER lead to similar weight loss [28,29,31]. Long-term results are highly variable, but in the long term, a plateau effect [31,38] or partial regain of lost weight [31,40,41,42] is observed. This suggests that the ability of patients to maintain long-term adherence to the recommendations and limit the effects of repeated cycles of weight loss and gain is more important than the CR or IER regimen itself.

In this context, adherence seems to be a key factor. In one study, adherence reached approximately 90% in the first months of intensive support, then dropped to 49% after limited contact with a dietitian [41]. This example does not allow for simple generalization, but it does illustrate that the effects of CR or IER depend not only on the mathematical energy deficit but also on behavioral burden and the availability of support. In clinical practice choice between CR and IER should consider the patient’s preferences, lifestyle, and barriers. Some individuals will benefit from a constant energy supply and a reduced decision making burden. Others will find it easier to accept clearly marked days or periods of greater restriction. In both cases, the structure of the intervention does not replace behavioral support, realistic expectations, and addressing psychological and environmental barriers.

Diet composition within CR and IER can modulate metabolic response, influence hunger perception, and facilitate or hinder energy deficit maintenance. Comparisons of LC and LF diets clearly illustrate the limitations of the available literature. LC was defined very broadly, ranging from less than 20% to approximately 35–40% of energy from carbohydrates, whereas LF included interventions with fat content below 30% of energy [43]. Studies with more stringent definitions for LC observed slightly greater weight loss in the initial phase than in the LF groups, but the differences were clinically small and limited to the initial phase [44]. In the 12-month study, individual weight changes in the LC and LF groups ranged widely, from significant weight loss to weight gain, despite comparable mean results for the studied groups, highlighting the wide variability in response to these strategies [45]. In a very short but tightly controlled metabolic study with isocaloric diets with different macronutrient distributions, there were no differences in weight loss [47]. Taken together, this supports the conclusion that, under controlled energy intake, neither pattern demonstrates a clear advantage, and that dietary selection should consider metabolic profile, preferences, and long-term feasibility. From a methodological perspective, more consistent definitions of LC and LF are needed to ensure that interventions classified as a given pattern do not differ significantly between studies [43,44].

This is even more evident in relation to KD variants. Significant short-term weight loss has been observed in selected populations, especially in the first months of the intervention [49,50]. However, metabolic studies conducted under conditions of balanced energy intake indicate that the KD is not superior to diets higher in carbohydrates in terms of fat loss [48]. Some studies have observed an increase in LDL cholesterol concentration [135], which may be important in the context of long-term cardiovascular risk [67]. This suggests that the KD may be a useful tool in specific clinical situations or in individuals who prefer this type of diet but requires careful monitoring of the lipid profile and the quality of consumed fat. Data on the effect of the ketogenic diet on hunger and satiety remain inconsistent, so potential benefits in this area should be interpreted with caution [51]. Its long-term advantage is currently unconfirmed and requires further well designed studies with long-term follow-up and systematic safety monitoring.

At the other extreme, the MedDiet has consistently demonstrated beneficial effects on glycemic control, insulin sensitivity, blood pressure, and lipid profile, particularly in individuals with type 2 diabetes and high cardiovascular risk [52,54]. Weight loss achieved with interventions based on this model was comparable to LC and more beneficial than LF [54]. However, results are mixed and have not always achieved clinically significant weight loss. The MedDiet combines high nutrient density, flexibility, and a favorable safety profile, making it an attractive baseline option for both CR and IER. This is particularly important in patients with obesity and coexisting metabolic disorders. In clinical practice, the MedDiet can serve as a framework for a diet that is then tailored to individual needs by modifying the intake of energy, protein, carbohydrates, and fats.

An important issue common to various dietary models is the preservation of lean body mass. Any intervention with an energy deficit is associated with some degree of muscle mass loss, depending on the intervention used [136]. The risk of lean body mass loss also becomes a challenge in the pharmacological treatment of obesity [15]. Available data indicate that a higher protein intake, reaching 1.2–2 g per kg of body weight per day, may limit the loss of lean body mass [57,136] and promote more favorable changes in body composition [56,58], especially when combined with resistance training [136]. In some studies, high protein diets led to slightly greater reductions in body weight and waist circumference than diets with a lower protein content at the same energy deficit [56,58], and in older adults, they limited the loss of lean body mass [57]. Improvements in selected metabolic parameters have also been observed in some populations [55,59], but the data are mixed. Concerns about kidney safety have not been confirmed in individuals with normal kidney function who consume at least 1.5 g per kg of body weight per day of protein [137]. Protein source appears to be more important for the prevention of chronic kidney disease, as higher intake of plant proteins has been associated with a lower risk of disease development, while high intake of red and processed meat has been associated with a higher risk of disease progression [138]. However, available data suggest that resistance training is the main factor protecting muscle mass during weight loss, and that higher protein intake serves as a supportive element that enhances the effect of exercise but does not replace the mechanical stimulus [128,129,136]. From a practical perspective, this approach can be integrated with various dietary patterns and energy restriction strategies, which may increase the flexibility of intervention planning.

A crucial element linking various nutritional strategies is the control of hunger and satiety. Weight loss leads to changes in the concentrations of appetite regulating hormones [139] and in the activity of central reward pathways [73,74]. Mathematical models have estimated that for every kilogram of body weight lost, appetite increases by approximately 100 kcal per day [65]. From a clinical perspective, this means that for many patients, the problem is not only the initial energy deficit, but also the increasing biological pressure favoring a return to previous eating patterns. A countermeasure strategy may be the deliberate use of dietary components that increase satiety for a given energy intake. Higher protein intake, ranging from 1.2 to 1.6 g per kilogram of body weight, has been repeatedly associated with reduced hunger, greater postprandial satiety, and spontaneous reduction in energy intake under ad libitum feeding conditions [76,77]. Increasing the share of low energy density products allowed for larger meal volumes with a relatively lower energy intake [80,81]. In one study, greater weight loss was observed among individuals who relied on lower energy density products, which facilitated energy restriction [46]. Incorporating approximately 30 g of fiber daily, particularly viscous fractions, was associated with better appetite control, reduced spontaneous energy intake, and favorable changes in the gut microbiota [82,83]. In addition, recent reviews have highlighted therapeutic modulation of the gut microbiota as a promising complementary target in obesity management. At present, however, the clinical evidence for microbiota directed strategies remains limited and heterogeneous, so their role should be regarded as exploratory and requires further high quality trials before specific recommendations can be made [140]. Sensory and structural characteristics of meals also play a role. Solid foods and those requiring longer chewing promoted greater satiety than liquids with the same energy value [85,86], while a slower eating pace and consuming meals without distractions facilitated the recognition of satiety signals and reduced overeating [87]. These mechanisms can be used regardless of whether the patient is following a MedDiet, LC, LF, or high protein diet because they involve the properties of meals rather than rigid macronutrient ratios.

Sleep also influences appetite regulation. Shortened sleep has been associated with decreased leptin levels, increased ghrelin levels, increased appetite, and a greater tendency to choose high calorie foods [115,117,118]. In adults, a sleep duration of approximately 7 to 9 h per night, with bedtime and wake up times as consistent as possible, is typically recommended [116]. In studies involving overweight individuals who slept less than 6.5 h per night, increased sleep duration was associated with a reduction in daily energy intake despite the lack of additional dietary recommendations [119]. A favorable sleep profile, including adequate length, regularity, quality, and efficiency, has been associated with greater weight and fat tissue loss [120] and a lower risk of weight regain [121]. On the other hand, weight loss may improve sleep quality and reduce the severity of obstructive sleep apnea, regardless of dietary composition [122,123]. This suggests that sleep should be considered an integral component of interventions aimed at weight loss and weight maintenance.

Physical activity is another key component of long-term obesity treatment strategies. Physical activity alone, even when consistent with public health recommendations, usually leads to modest weight loss [124,126], and the response to exercise varies significantly among individuals [126]. More pronounced and predictable effects have been observed when physical activity is combined with dietary changes [12,132]. Against this background, epidemiological observations are particularly concerning, showing that over 30% of adults do not meet the minimum recommendations for physical activity, and this percentage exceeds 40% among older adults [141]. This means that a significant proportion of patients begin obesity treatment from a very low baseline, which influences the selection of realistic exercise recommendations.

In groups of individuals who maintained long-term weight loss, regular physical activity was reported significantly more frequently [12,125,132]. The most frequently reported form of activity was walking, supplemented by other forms of moderate-intensity exercise [132]. Population based data indicate that even small increases in the number of steps per day are associated with a lower risk of premature death, with the strongest effects observed at approximately 7000 to 10,000 steps per day [142,143]. The best results in terms of fat reduction and protection of lean body mass were observed in programs combining aerobic training with resistance training [129]. Physical activity also improves blood pressure, glycemic control, and lipid profile, regardless of changes in body weight [130,131], and may support appetite regulation through changes in leptin and ghrelin levels [131]. Additionally, it is associated with a reduction in symptoms of depression, anxiety, and chronic stress, which is important for patients with obesity, who often experience psychological distress [130]. From a clinical and practical perspective, not every intervention needs to begin with a high volume exercise. For individuals with numerous contraindications or barriers to more intense exercise, regular walking alone and gradually increasing the number of steps may be a reasonable first goal, supporting both cardiometabolic health and long-term maintenance of weight loss. However, it is also prudent to incorporate resistance training due to its unique benefits in protecting lean body mass.

Psychological and behavioral factors also significantly influence the outcomes of dietary treatment. Emotional eating, difficulties regulating emotions, episodes of loss of control overeating, and unplanned snacking are more frequently reported by individuals with obesity and may hinder adherence to recommendations [88,89,90]. Emotional suppression has been associated with a higher risk of psychological problems, which may impair the ability to achieve lasting behavior change [91]. On the other hand, interventions targeting emotion regulation, including mindfulness based approaches and techniques that enhance response control to food stimuli, have been associated with improved impulse control and greater consistency of eating behaviors with long-term goals [92,93]. Some studies also emphasize the role of body image. Improved body acceptance was associated with more stable maintenance of weight control behaviors, especially in individuals with low initial body image satisfaction [94]. Psychological and behavioral interventions generally produce small to moderate additional effects on weight loss but appear crucial for long term weight loss maintenance by enhancing self-regulation, body image and habit formation. Given the heterogeneous and often small-scale evidence base, these strategies are best viewed not as stand alone weight loss treatments but as key modulators of adherence and relapse prevention, with high clinical relevance. These mechanisms also correspond to core components of the COM-B model. Processes such as emotion regulation, impulse control, body acceptance and habit formation reflect psychological capability and reflective motivation within this framework. Evidence from recent applications of COM-B in individuals with overweight and obesity suggests that self-efficacy plays an important role in facilitating these processes [144], and may support the implementation and maintenance of behavioral change.

Motivation, habits, and cognitive load constitute another group of determinants. Many studies have observed a gradual decline in motivation, diet fatigue [95], and a slower rate of weight loss [96]. These phenomena correspond well with the results of numerous studies on weight loss, in which an initial weight loss is followed by a plateau or partial weight regain, despite formally unchanged intervention assumptions [12]. Unrealistic expectations regarding the rate of weight loss have been associated with a higher risk of frustration and discontinuation of the intervention [97]. A rigidly restrictive approach to dieting and perceiving weight loss as a short-term phase rather than a permanent lifestyle change has been associated with a higher risk of relapse to previous eating patterns [99,100]. Data on self-regulation strategies indicate that regular behavior monitoring, planning, and flexible adjustments are more frequently observed in individuals achieving long-term results [94]. Interventions aimed at shaping habits promoted more repetitive behaviors supporting weight control [101], and elements reducing the number of daily dietary decisions, such as a consistent meal structure, may be perceived as easier to maintain [102].

Social and environmental factors also play a significant role. Weight related stigma has been associated with higher stress levels and difficulties in maintaining health promoting behaviors [103], while negative beliefs about one’s body weight have been associated with avoiding physical activity and a higher risk of depressive symptoms [104]. Limited support from family and social networks has been associated with a higher likelihood of intervention discontinuation [105]. Studies examining various forms of support have shown that group interventions are associated with greater engagement and regularity of behaviors [93,106], while in individual interventions, the quality of the relationship between the specialist and the participant correlated with better adherence to recommendations [107]. Programs delivered exclusively digitally were more likely to have less long-lasting effects, while hybrid models with at least partial in-person contact demonstrated better adherence and a higher likelihood of maintaining results [108,109]. Digital and telehealth elements have considerable potential due to their scalability but require further refinement to better support long term adherence. Evidence from studies of the food environment indicates that the affordability and availability of minimally processed foods, as well as the local density of outlets selling predominantly ultra processed products, are associated with obesity risk [145]. These structural factors can constrain the extent to which individual patients are able to implement and maintain dietary recommendations, which underscores that long term adherence is shaped not only by personal motivation and skills but also by broader environmental and policy level determinants.

Additional insight is provided by data from registries and observational studies describing individuals who maintain long-term weight loss. This population was more likely to systematically monitor their weight and behaviors, engage in regular physical activity, and maintain a consistent pattern of eating habits [12,132,133]. Diets tended to be moderate in energy density and predominantly minimally processed [132,133]. Preventive strategies, such as meal planning, preparing food in advance, using shopping lists, and limiting the availability of high-calorie foods in the environment, were more frequently reported [132,133,134]. A gradual shift in health mindset was also described. These individuals were more likely to perceive themselves as more active and health oriented, which facilitated the incorporation of supportive behaviors into their daily lives and the flexibility to respond to periodic deviations [133]. Social support from family, loved ones, and professionals was associated with greater consistency in maintaining their weight loss [133,134]. These data suggest that long-term success is more likely to result from repeated practice of simple behaviors and gradual change in health identity than from selecting a single optimal dietary pattern.

From a clinical perspective, the results of this review indicate that the choice of obesity treatment strategy should be primarily focused on identifying the predominant barriers in each patient and adapting their dietary pattern, energy structure, recommendations for physical activity, sleep, and psychological support to address them. For individuals whose primary concern is intense hunger and difficulty controlling appetite, prioritizing these issues should be a priority. If psychological barriers, such as emotional eating, rigid patterns of thinking about food, or severe depressive symptoms, are prevalent, it is reasonable to consider incorporating psychological interventions based on cognitive behavioral therapy, acceptance and commitment approaches, and mindfulness-based interventions, with parallel work on stigma and social support. In cases of significant sleep disturbances or chronic sleep deprivation, priority should be given to regulating the circadian rhythm and increasing sleep duration, which can facilitate both appetite control and emotion regulation. In patients at high cardiovascular risk, basing the intervention on a MedDiet with an adequately high protein intake and regular physical activity is particularly warranted. Regardless of the dietary model used, it is crucial to plan in parallel to protect lean body mass through adequate protein supply and resistance training, especially in older people and patients receiving pharmacological treatment.

The available literature is characterized by significant limitations. Many studies combine overweight individuals with varying degrees of obesity, making it difficult to draw conclusions specific to the population with obesity. Definitions of LC and LF diets are inconsistent, and interventions often encompass simultaneous energy, quality, and behavioral changes, making it difficult to isolate the impact of individual components. Most studies comparing CR and IER last less than 12 months, and adherence is measured irregularly or indirectly. Detailed measures of cognitive load, perceived protocol burden, and quality of life are rarely included. This limits the ability to fully assess the feasibility of individual strategies in everyday life. In many studies, the main results are presented as group mean values, which masks significant variability in individual weight trajectories. Most data on appetite regulation mechanisms and the role of sleep and physical activity are based on short-term interventions, making extrapolation to long-term weight maintenance difficult. Together, these sources of methodological heterogeneity substantially constrain direct comparability between interventions and limit the generalizability of conclusions to routine clinical practice.

The lack of a formal assessment of methodological quality and risk of bias represents an additional limitation of this narrative review. The included studies differed substantially in design, intervention definitions, comparators, duration, and outcome measures. This degree of heterogeneity prevented the use of a single coherent appraisal tool and therefore limited the possibility of directly comparing the strength of evidence across studies. As a result, the conclusions should be interpreted with appropriate caution. However, synthesizing these diverse findings within a unified conceptual framework remains clinically valuable, because it reflects the complexity of real-world obesity management and highlights consistent patterns that emerge despite methodological variability.

## 5. Conclusions

Long term obesity management depends on the integration of nutritional, behavioral, psychological and lifestyle components rather than on any single dietary strategy. Evidence shows that clinically meaningful weight loss can be achieved using various energy restriction models, yet the durability of outcomes is shaped primarily by appetite regulation, sleep, physical activity, emotional and cognitive processes, social context, and environmental constraints. Protecting lean body mass, reducing behavioral burden, supporting self-regulation, and addressing individual barriers represent core elements of sustainable care.

Future research should include longer follow-up, standardized reporting of adherence and behavioral load, and more precise identification of patient profiles that benefit from specific combinations of nutritional, behavioral and pharmacological strategies. A multidimensional approach appears essential for translating short term weight loss into durable long-term outcomes.

## Figures and Tables

**Table 1:** Comparison of intermittent energy restriction (IER) effectiveness in weight loss compared to continuous energy restriction (CR) diets and ad libitum interventions.

Source	Number of Included Studies	Population	Type of IER	Comparison	Duration of the Intervention (Weeks)	Short-Term Weight Loss (kg)	Long-Term Weight Loss (kg)	Other Conclusions
Silverii (2023) [26]	9 RCT	Women and men BMI ≥ 30	TRE and PF	CR and ad libitum diet	8–56 weeks	On average, −2.05 kg in favor of IER in 8–12 weeks	Average −2.73 kg in favor of IER in 24–32 weeks. No difference in 40–56 weeks	-
Xie (2024) [27]	20 RCT	Women and men BMI ≥ 25	TRE	CR and ad libitum	6–52 weeks	≤39 weeks average loss −2.13 kg in favor of IER	≥39 weeks average loss −2.02 kg in favor of IER	Combined analysis showed an average of −2.11 kg in favor of IER. Feeding window 6–8 h, average loss −2.73 kg, feeding window 10–12 h, no difference compared to the control group
Ezzati (2023) [28]	13 RCT	Women and men BMI ≥ 25	TRE, ADF, PF	CR	8–52 weeks	no differences between groups	no differences between groups	Better weight loss results were seen in studies in which food was delivered to the subjects.
He (2021) [29]	11 RCT	Women and men BMI ≥ 25	AFD, 5:2	CR	8–48 weeks	≤12 weeks better loss on average by −1.66 kg in favor of IER	no differences between groups	The better loss in the short term was probably due to spontaneous restriction of calorie intake on feeding days.
Yao (2024) [30]	9 RCT	Women and men > 40 years with BMI > 24	TRE, PF, ADF, religious posts	ad libitum	6–12 weeks	better weight loss on average by −2.05 kg in favor of IER	no data	Subgroup analysis showed an average loss of −1.63 kg in individuals with a BMI of 25–34.9 and an average loss of −4.46 kg in individuals with a BMI of >35
Enríquez Guerrero (2021) [31]	18 RCT	Women and men BMI ≥ 25	ADF, PF	CR	8–48 weeks	no differences between groups	no differences between groups	Little long-term work indicates a plateau of intervention or weight regain.
Wei (2022) [32]	27 RCT	Women and men BMI ≥ 25	TRE, ADF, PF, WOWO	CR or ad libitum	7.5–52 weeks	better results in favor of IER compared to ad libitum, no differences compared to CR	no difference compared to ad libitum and CR	-
Patikorn (2021) [33]	11 meta-analyses	Women and men	TRE, PF, ADF	CR or ad libitum	4–48 weeks	better results in favor of IER ≤ 24 weeks	noticeable plateau phase	MADF and PF (5:2) were the only IER types that were associated with statistically significant weight loss of more than 5% in adults with overweight and obesity.

Legend: TRE—time restricting eating, PF—periodic fasting, ADF—alternate day fasting, MADF—modified alternate-day fasting, CR—continuous calorie restriction, IER—intermittent energy restriction, WOWO—week-on-week-of.

## Data Availability

No new data were created or analyzed in this study. Data sharing is not applicable to this article.

## References

[1] Chamarthi V.S., Daley S.F. (2025). Comprehensive Assessment and Diagnosis of Metabolic and Biomechanical Complications in Obesity. StatPearls.

[2] Masood B., Moorthy M. (2023). Causes of obesity: A review. Clin. Med..

[3] Anekwe C.V., Jarrell A.R., Townsend M.J., Gaudier G.I., Hiserodt J.M., Stanford F.C. (2020). Socioeconomics of Obesity. Curr. Obes. Rep..

[4] Lin X., Li H. (2021). Obesity: Epidemiology, Pathophysiology, and Therapeutics. Front. Endocrinol..

[5] Sørensen T.I.A., Martinez A.R., Jørgensen T.S.H., Eckel J., Clément K.R. (2022). Epidemiology of Obesity. From Obesity to Diabetes.

[6] World Obesity Federation (2023). World Obesity Atlas. https://data.worldobesity.org/publications/?cat=19.

[7] Westbury S., Oyebode O., van Rens T., Barber T.M. (2023). Obesity Stigma: Causes, Consequences, and Potential Solutions. Curr. Obes. Rep..

[8] Peeters A., Barendregt J.J., Willekens F., MacKenbach J.P., Mamun A., Bonneux L. (2003). Obesity in adulthood and its consequences for life expectancy: A life-table analysis. Ann. Intern. Med..

[9] Garvey W.T., Mechanick J.I., Brett E.M., Garber A.J., Hurley D.L., Jastreboff A.M., Nadolsky K., Pessah-Pollack R., Plodkowski R., Reviewers of the AACE/ACE Obesity Clinical Practice Guidelines (2016). American Association of Clinical Endocrinologists and American College of Endocrinology Comprehensive Clinical Practice Guidelines for Medical Care of Patients with Obesity. Endocr. Pract..

[10] Juul-Hindsgaul N., Alalwani Z., Boylan A., Hartmann-Boyce J., Nunan D. (2024). Defining success in adult obesity management: A systematic review and framework synthesis of clinical practice guidelines. Clin. Obes..

[11] Anderson J.W., Konz E.C., Frederich R.C., Wood C.L. (2001). Long-term weight-loss maintenance: A meta-analysis of US studies. Am. J. Clin. Nutr..

[12] Hall K.D., Kahan S. (2018). Maintenance of Lost Weight and Long-Term Management of Obesity. Med. Clin. N. Am..

[13] Lu J., Liu P., Cai M., Lv T., Zhang M., Yin K., Cheng J., Zhang G. (2025). Recent progress in the pharmacotherapy for obesity. Eur. J. Pharmacol..

[14] Rubino D., Abrahamsson N., Davies M., Hesse D., Greenway F.L., Jensen C., Lingvay I., Mosenzon O., Rosenstock J., Rudofsky G. (2021). Effect of Continued Weekly Subcutaneous Semaglutide vs Placebo on Weight Loss Maintenance in Adults with Overweight or Obesity: The STEP 4 Randomized Clinical Trial. JAMA.

[15] Neeland I.J., Linge J., Birkenfeld A.L. (2024). Changes in lean body mass with glucagon-like peptide-1-based therapies and mitigation strategies. Diabetes Obes. Metab..

[16] Kim J.Y. (2021). Optimal Diet Strategies for Weight Loss and Weight Loss Maintenance. J. Obes. Metab. Syndr..

[17] Hall K.D., Farooqi I.S., Friedman J.M., Klein S., Loos R.J.F., Mangelsdorf D.J., O’rAhilly S., Ravussin E., Redman L.M., Ryan D.H. (2022). The energy balance model of obesity: Beyond calories in, calories out. Am. J. Clin. Nutr..

[18] Muscogiuri G., El Ghoch M., Colao A., Hassapidou M., Yumuk V., Busetto L. (2021). European Guidelines for Obesity Management in Adults with a Very Low-Calorie Ketogenic Diet: A Systematic Review and Meta-Analysis. Obes. Facts.

[19] Kloecker D.E., Zaccardi F., Baldry E., Davies M.J., Khunti K., Webb D.R. (2019). Efficacy of low- and very-low-energy diets in people with type 2 diabetes mellitus: A systematic review and meta-analysis of interventional studies. Diabetes Obes. Metab..

[20] Lane M., Howland G., West M., Hockey M., Marx W., Loughman A., O’hEly M., Jacka F., Rocks T. (2020). The effect of ultra-processed very low-energy diets on gut microbiota and metabolic outcomes in individuals with obesity: A systematic literature review. Obes. Res. Clin. Pract..

[21] Castellana M., Conte E., Cignarelli A., Perrini S., Giustina A., Giovanella L., Giorgino F., Trimboli P. (2020). Efficacy and safety of very low calorie ketogenic diet (VLCKD) in patients with overweight and obesity: A systematic review and meta-analysis. Rev. Endocr. Metab. Disord..

[22] Parretti H.M., Jebb S.A., Johns D.J., Lewis A.L., Christian-Brown A.M., Aveyard P. (2016). Clinical effectiveness of very-low-energy diets in the management of weight loss: A systematic review and meta-analysis of randomized controlled trials. Obes. Rev..

[23] Trouwborst I., Goossens G.H., Astrup A., Saris W.H.M., Blaak E.E. (2021). Sexual Dimorphism in Body Weight Loss, Improvements in Cardiometabolic Risk Factors and Maintenance of Beneficial Effects 6 Months after a Low-Calorie Diet: Results from the Randomized Controlled DiOGenes Trial. Nutrients.

[24] Christensen P., Larsen T.M., Westerterp-Plantenga M., Macdonald I., Martinez J.A., Handjiev S., Poppitt S., Hansen S., Ritz C., Astrup A. (2018). Men and women respond differently to rapid weight loss: Metabolic outcomes of a multi-centre intervention study after a low-energy diet in 2500 overweight, individuals with pre-diabetes (PREVIEW). Diabetes Obes. Metab..

[25] Seimon R.V., Wild-Taylor A.L., Keating S.E., McClintock S., Harper C., Gibson A.A., Johnson N.A., Fernando H.A., Markovic T.P., Center J.R. (2019). Effect of Weight Loss via Severe vs Moderate Energy Restriction on Lean Mass and Body Composition Among Postmenopausal Women with Obesity: The TEMPO Diet Randomized Clinical Trial. JAMA Netw. Open.

[26] Silverii G.A., Cresci B., Benvenuti F., Santagiuliana F., Rotella F., Mannucci E. (2023). Effectiveness of intermittent fasting for weight loss in individuals with obesity: A meta-analysis of randomized controlled trials. Nutr. Metab. Cardiovasc. Dis..

[27] Xie Y., Zhou K., Shang Z., Bao D., Zhou J. (2024). The Effects of Time-Restricted Eating on Fat Loss in Adults with Overweight and Obese Depend Upon the Eating Window and Intervention Strategies: A Systematic Review and Meta-Analysis. Nutrients.

[28] Ezzati A., Rosenkranz S.K., Phelan J., Logan C. (2023). The Effects of Isocaloric Intermittent Fasting vs Daily Caloric Restriction on Weight Loss and Metabolic Risk Factors for Noncommunicable Chronic Diseases: A Systematic Review of Randomized Controlled or Comparative Trials. J. Acad. Nutr. Diet..

[29] He S., Wang J., Zhang J., Xu J. (2021). Intermittent Versus Continuous Energy Restriction for Weight Loss and Metabolic Improvement: A Meta-Analysis and Systematic Review. Obesity.

[30] Yao K., Su H., Cui K., Gao Y., Xu D., Wang Q., Ha Z., Zhang T., Chen S., Liu T. (2024). Effectiveness of an intermittent fasting diet versus regular diet on fat loss in overweight and obese middle-aged and elderly people without metabolic disease: A systematic review and meta-analysis of randomized controlled trials. J. Nutr. Health Aging.

[31] Enríquez Guerrero A., Martín I.S.M., Vilar E.G., Martín M.A.C. (2021). Effectiveness of an intermittent fasting diet versus continuous energy restriction on anthropometric measurements, body composition and lipid profile in overweight and obese adults: A meta-analysis. Eur. J. Clin. Nutr..

[32] Wei X., Cooper A., Lee I., Cernoch C.A., Huntoon G., Hodek B., Christian H., Chao A.M. (2022). Intermittent Energy Restriction for Weight Loss: A Systematic Review of Cardiometabolic, Inflammatory and Appetite Outcomes. Biol. Res. Nurs..

[33] Patikorn C., Roubal K., Veettil S.K., Chandran V., Pham T., Lee Y.Y., Giovannucci E.L., Varady K.A., Chaiyakunapruk N. (2021). Intermittent Fasting and Obesity-Related Health Outcomes: An Umbrella Review of Meta-analyses of Randomized Clinical Trials. JAMA Netw. Open.

[34] Hwalla N., Jaafar Z. (2021). Dietary Management of Obesity: A Review of the Evidence. Diagnostics.

[35] Koliaki C., Spinos T., Spinou M., Brinia M.-E., Mitsopoulou D., Katsilambros N. (2018). Defining the optimal dietary approach for safe, effective and sustainable weight loss in overweight and obese adults. Healthcare.

[36] Jovanović G.K., Mrakovcic-Sutic I., Žeželj S.P., Šuša B., Rahelić D., Majanović S.K. (2020). The Efficacy of an Energy-Restricted Anti-Inflammatory Diet for the Management of Obesity in Younger Adults. Nutrients.

[37] Cheng C.-C., Hsu C.-Y., Liu J.-F. (2018). Effects of dietary and exercise intervention on weight loss and body composition in obese postmenopausal women: A systematic review and meta-analysis. Menopause.

[38] Sundfør T.M., Svendsen M., Tonstad S. (2018). Effect of intermittent versus continuous energy restriction on weight loss, maintenance and cardiometabolic risk: A randomized 1-year trial. Nutr. Metab. Cardiovasc. Dis..

[39] Carter S., Clifton P.M., Keogh J.B. (2018). Effect of Intermittent Compared with Continuous Energy Restricted Diet on Glycemic Control in Patients with Type 2 Diabetes: A Randomized Noninferiority Trial. JAMA Netw. Open.

[40] Schübel R., Nattenmüller J., Sookthai D., Nonnenmacher T., Graf M.E., Riedl L., Schlett C.L., von Stackelberg O., Johnson T., Nabers D. (2018). Effects of intermittent and continuous calorie restriction on body weight and metabolism over 50 wk: A randomized controlled trial. Am. J. Clin. Nutr..

[41] Carter S., Clifton P.M., Keogh J.B. (2019). The effect of intermittent compared with continuous energy restriction on glycaemic control in patients with type 2 diabetes: 24-month follow-up of a randomised noninferiority trial. Diabetes Res. Clin. Pract..

[42] Liu D., Huang Y., Huang C., Yang S., Wei X., Zhang P., Guo D., Lin J., Xu B., Li C. (2022). Calorie Restriction with or without Time-Restricted Eating in Weight Loss. N. Engl. J. Med..

[43] Ge L., Sadeghirad B., Ball G.D.C., Da Costa B.R., Hitchcock C.L., Svendrovski A., Kiflen R., Quadri K., Kwon H.Y., Karamouzian M. (2020). Comparison of dietary macronutrient patterns of 14 popular named dietary programmes for weight and cardiovascular risk factor reduction in adults: Systematic review and network meta-analysis of randomised trials. BMJ.

[44] Mansoor N., Vinknes K.J., Veierød M.B., Retterstøl K. (2016). Effects of low-carbohydrate diets v. low-fat diets on body weight and cardiovascular risk factors: A meta-analysis of randomised controlled trials. Br. J. Nutr..

[45] Gardner C.D., Trepanowski J.F., Del Gobbo L.C., Hauser M.E., Rigdon J., Ioannidis J.P., Desai M., King A.C. (2018). Effect of Low-Fat vs Low-Carbohydrate Diet on 12-Month Weight Loss in Overweight Adults and the Association with Genotype Pattern or Insulin Secretion: The DIETFITS Randomized Clinical Trial. JAMA.

[46] Hauser M.E., Hartle J.C., Landry M.J., Fielding-Singh P., Shih C.W., Qin F., Rigdon J., Gardner C.D. (2024). Association of dietary adherence and dietary quality with weight loss success among those following low-carbohydrate and low-fat diets: A secondary analysis of the DIETFITS randomized clinical trial. Am. J. Clin. Nutr..

[47] Hall K.D., Bemis T., Brychta R., Chen K.Y., Courville A., Crayner E.J., Goodwin S., Guo J., Howard L., Knuth N.D. (2015). Calorie for Calorie, Dietary Fat Restriction Results in More Body Fat Loss than Carbohydrate Restriction in People with Obesity. Cell Metab..

[48] Hall K.D., Chen K.Y., Guo J., Lam Y.Y., Leibel R.L., Mayer L.E., Reitman M.L., Rosenbaum M., Smith S.R., Walsh B.T. (2016). Energy expenditure and body composition changes after an isocaloric ketogenic diet in overweight and obese men. Am. J. Clin. Nutr..

[49] Xing N.-N., Ren F., Yang H. (2024). Effects of ketogenic diet on weight loss parameters among obese or overweight patients with polycystic ovary syndrome: A systematic review and meta-analysis of randomized controlled trails. Food Nutr. Res..

[50] Mohorko N., Černelič-Bizjak M., Poklar-Vatovec T., Grom G., Kenig S., Petelin A., Jenko-Pražnikar Z. (2019). Weight loss, improved physical performance, cognitive function, eating behavior, and metabolic profile in a 12-week ketogenic diet in obese adults. Nutr. Res..

[51] Baylie T., Ayelgn T., Tiruneh M., Tesfa K. (2024). Effect of Ketogenic Diet on Obesity and Other Metabolic Disorders: Narrative Review. Diabetes Metab. Syndr. Obes..

[52] Zheng X., Zhang W., Wan X., Lv X., Lin P., Si S., Xue F., Wang A., Cao Y. (2024). The effects of Mediterranean diet on cardiovascular risk factors, glycemic control and weight loss in patients with type 2 diabetes: A meta-analysis. BMC Nutr..

[53] Currenti W., Losavio F., Quiete S., Alanazi A.M., Messina G., Polito R., Ciolli F., Zappalà R.S., Galvano F., Cincione R.I. (2024). Comparative Evaluation of a Low-Carbohydrate Diet and a Mediterranean Diet in Overweight/Obese Patients with Type 2 Diabetes Mellitus: A 16-Week Intervention Study. Nutrients.

[54] Mancini J.G., Filion K.B., Atallah R., Eisenberg M.J. (2016). Systematic Review of the Mediterranean Diet for Long-Term Weight Loss. Am. J. Med..

[55] Flores-Hernández M.N., Martínez-Coria H., López-Valdés H.E., Arteaga-Silva M., Arrieta-Cruz I., Gutiérrez-Juárez R. (2024). Efficacy of a High-Protein Diet to Lower Glycemic Levels in Type 2 Diabetes Mellitus: A Systematic Review. Int. J. Mol. Sci..

[56] Lauran M., Jafari A.M., Ali K.M., Hosseini M. (2024). The Effects of High Protein Intake on Cardiovascular Risk Factors and Weight Loss in Low Caloric Diets in Obese Adults: A Systematic Review. J. Biostat. Epidemiol..

[57] Wright C.S., Zhou J., Sayer R.D., Kim J.E., Campbell W.W. (2018). Effects of a High-Protein Diet Including Whole Eggs on Muscle Composition and Indices of Cardiometabolic Health and Systemic Inflammation in Older Adults with Overweight or Obesity: A Randomized Controlled Trial. Nutrients.

[58] Campos-Nonato I., Hernandez L., Barquera S. (2017). Effect of a High-Protein Diet Versus Standard-Protein Diet on Weight Loss and Biomarkers of Metabolic Syndrome: A Randomized Clinical Trial. Obes. Facts.

[59] Yu Z., Nan F., Wang L., Jiang H., Chen W., Jiang Y. (2020). Effects of high-protein diet on glycemic control, insulin resistance and blood pressure in type 2 diabetes: A systematic review and meta-analysis of randomized controlled trials. Clin. Nutr..

[60] Nunes C.L., Casanova N., Francisco R., Bosy-Westphal A., Hopkins M., Sardinha L.B., Silva A.M. (2022). Does adaptive thermogenesis occur after weight loss in adults? A systematic review. Br. J. Nutr..

[61] Martins C., Roekenes J., Salamati S., Gower B.A., Hunter G.R. (2020). Metabolic adaptation is an illusion, only present when participants are in negative energy balance. Am. J. Clin. Nutr..

[62] Martins C., Gower B.A., Hunter G.R. (2022). Metabolic adaptation delays time to reach weight loss goals. Obesity.

[63] Fothergill E., Guo J., Howard L., Kerns J.C., Knuth N.D., Brychta R., Chen K.Y., Skarulis M.C., Walter M., Walter P.J. (2016). Persistent metabolic adaptation 6 years after “The Biggest Loser” competition. Obesity.

[64] Byrne N.M., Sainsbury A., King N.A., Hills A.P., Wood R.E. (2018). Intermittent energy restriction improves weight loss efficiency in obese men: The MATADOR study. Int. J. Obes..

[65] Polidori D., Sanghvi A., Seeley R.J., Hall K.D. (2016). How Strongly Does Appetite Counter Weight Loss? Quantification of the Feedback Control of Human Energy Intake. Obesity.

[66] Iepsen E.W., Lundgren J., Holst J.J., Madsbad S., Torekov S.S. (2016). Successful weight loss maintenance includes long-term increased meal responses of GLP-1 and PYY3–36. Eur. J. Endocrinol..

[67] Tong J., Heianza Y., Schur E.A., Sacks F., Qi L., Boyko E.J. (2023). FRI074 Rise in Fasting Ghrelin Level in Response to Weight Loss Predicts Future Weight Regain in the POUNDS LOST Trial. J. Endocr. Soc..

[68] Lean M.E.J., Malkova D. (2016). Altered gut and adipose tissue hormones in overweight and obese individuals: Cause or consequence?. Int. J. Obes..

[69] Chen H.-D., Wu D.-A., Jia-Sian H., Yi-Maun S., Jer-Chuan L., Bang-Gee H. (2017). Positive correlation of serum leptin levels with obesity and metabolic syndrome in patients with type 2 diabetes mellitus. Int. J. Clin. Exp. Pathol..

[70] Rashad N.M., Sayed S.E., Sherif M.H., Sitohy M.Z. (2019). Effect of a 24-week weight management program on serum leptin level in correlation to anthropometric measures in obese female: A randomized controlled clinical trial. Diabetes Metab. Syndr..

[71] Kempf K., Röhling M., Banzer W., Braumann K.M., Halle M., Schaller N., McCarthy D., Predel H.G., Schenkenberger I., Tan S. (2022). Early and Strong Leptin Reduction is Predictive for Long-Term Weight Loss During High-Protein, Low-Glycaemic Meal Replacement—A Subanalysis of the Randomised-Controlled ACOORH Trial. Nutrients.

[72] Verdejo-Román J., Vilar-López R., Navas J.F., Soriano-Mas C., Verdejo-García A. (2017). Brain reward system’s alterations in response to food and monetary stimuli in overweight and obese individuals: Food and Monetary Processing in Overweight and Obesity. Hum. Brain Mapp..

[73] Nock N.L., Jiang H., Borato L., Alberts J., Dimitropoulos A. (2020). Insights to the neural response to food cues in class III compared with class I and II obese adults using a sample of endometrial cancer survivors seeking weight loss. Nutr. Diabetes.

[74] Devoto F., Zapparoli L., Bonandrini R., Berlingeri M., Ferrulli A., Luzi L., Banfi G., Paulesu E. (2018). Hungry brains: A meta-analytical review of brain activation imaging studies on food perception and appetite in obese individuals. Neurosci. Biobehav. Rev..

[75] Hansen T.T., Mead B.R., García-Gavilán J.F., Korndal S.K., Harrold J.A., Camacho-Barcía L., Ritz C., Christiansen P., Salas-Salvadó J., Hjorth M.F. (2019). Is reduction in appetite beneficial for body weight management in the context of overweight and obesity? Yes, according to the SATIN (Satiety Innovation) study. J. Nutr. Sci..

[76] Leidy H.J., Clifton P.M., Astrup A., Wycherley T.P., Westerterp-Plantenga M.S., Luscombe-Marsh N.D., Woods S.C., Mattes R.D. (2015). The role of protein in weight loss and maintenance. Am. J. Clin. Nutr..

[77] De Carvalho K.M.B., Pizato N., Botelho P.B., Dutra E.S., Gonçalves V.S.S. (2020). Dietary protein and appetite sensations in individuals with overweight and obesity: A systematic review. Eur. J. Nutr..

[78] Raubenheimer D., Simpson S.J. (2019). Protein Leverage: Theoretical Foundations and Ten Points of Clarification. Obesity.

[79] Moon J., Koh G. (2020). Clinical Evidence and Mechanisms of High-Protein Diet-Induced Weight Loss. J. Obes. Metab. Syndr..

[80] Klos B., Cook J., Crepaz L., Weiland A., Zipfel S., Mack I. (2023). Impact of energy density on energy intake in children and adults: A systematic review and meta-analysis of randomized controlled trials. Eur. J. Nutr..

[81] Iversen K.N., Carlsson F., Andersson A., Michaëlsson K., Langton M., Risérus U., Hellström P.M., Landberg R. (2021). A hypocaloric diet rich in high fiber rye foods causes greater reduction in body weight and body fat than a diet rich in refined wheat: A parallel randomized controlled trial in adults with overweight and obesity (the RyeWeight study). Clin. Nutr. ESPEN.

[82] Jovanovski E., Mazhar N., Komishon A., Khayyat R., Li D., Mejia S.B., Khan T., Jenkins A.L., Smircic-Duvnjak L., Sievenpiper J.L. (2020). Can dietary viscous fiber affect body weight independently of an energy-restrictive diet? A systematic review and meta-analysis of randomized controlled trials. Am. J. Clin. Nutr..

[83] Tremblay A., Clinchamps M., Pereira B., Courteix D., Lesourd B., Chapier R., Obert P., Vinet A., Walther G., Chaplais E. (2020). Dietary Fibres and the Management of Obesity and Metabolic Syndrome: The RESOLVE Study. Nutrients.

[84] Christensen L., Vuholm S., Roager H.M., Nielsen D.S., Krych L., Kristensen M., Astrup A., Hjorth M.F. (2019). Prevotella Abundance Predicts Weight Loss Success in Healthy, Overweight Adults Consuming a Whole-Grain Diet Ad Libitum: A Post Hoc Analysis of a 6-Wk Randomized Controlled Trial. J. Nutr..

[85] Miquel-Kergoat S., Azais-Braesco V., Burton-Freeman B., Hetherington M.M. (2015). Effects of chewing on appetite, food intake and gut hormones: A systematic review and meta-analysis. Physiol. Behav..

[86] Krop E.M., Hetherington M.M., Nekitsing C., Miquel S., Postelnicu L., Sarkar A. (2018). Influence of oral processing on appetite and food intake—A systematic review and meta-analysis. Appetite.

[87] van Meer F., de Vos F., Hermans R.C.J., Peeters P.A., van Dillen L.F. (2022). Daily distracted consumption patterns and their relationship with BMI. Appetite.

[88] Ford T., Lee H., Jeon M. (2017). The emotional eating and negative food relationship experiences of obese and overweight adults. Soc. Work Health Care.

[89] Mason A.E., Epel E.S., Aschbacher K., Lustig R.H., Acree M., Kristeller J., Cohn M., Dallman M., Moran P.J., Bacchetti P. (2016). Reduced reward-driven eating accounts for the impact of a mindfulness-based diet and exercise intervention on weight loss: Data from the SHINE randomized controlled trial. Appetite.

[90] Micanti F., Iasevoli F., Cucciniello C., Costabile R., Loiarro G., Pecoraro G., Pasanisi F., Rossetti G.L., Galletta D. (2017). The relationship between emotional regulation and eating behaviour: A multidimensional analysis of obesity psychopathology. Eat. Weight Disord. Stud. Anorex. Bulim. Obes..

[91] Andrei F., Nuccitelli C., Mancini G., Reggiani G.M., Trombini E. (2018). Emotional intelligence, emotion regulation and affectivity in adults seeking treatment for obesity. Psychiatry Res..

[92] Carrière K., Khoury B., Günak M.M., Knäuper B. (2018). Mindfulness-based interventions for weight loss: A systematic review and meta-analysis. Obes. Rev..

[93] Kaur T., Ranjan P., Kaloiya G.S., Bhatia H., Prakash B., Singh A., Sarkar S., Jadon R.S., Jorwal P., Baitha U. (2024). Effectiveness of cognitive retraining intervention on weight loss and lifestyle-related behaviours among adults: A systematic review and meta-analysis. Diabetes Metab. Syndr..

[94] Teixeira P.J., Carraça E.V., Marques M.M., Rutter H., Oppert J.-M., De Bourdeaudhuij I., Lakerveld J., Brug J. (2015). Successful behavior change in obesity interventions in adults: A systematic review of self-regulation mediators. BMC Med..

[95] Nichols R.M. (2017). Being Able to Be Stable: A Mixed Methods Analysis of Behavior and Motivation Predictors That Promote Long-Term Weight-Loss Maintenance. Ph.D. Dissertation.

[96] Dicker D., Alfadda A.A., Coutinho W., Cuevas A., Halford J.C., Hughes C.A., Iwabu M., Kang J.-H., Nawar R., Reynoso R. (2021). Patient motivation to lose weight: Importance of healthcare professional support, goals and self-efficacy. Eur. J. Intern. Med..

[97] Poulimeneas D., Anastasiou C.A., Kokkinos A., Panagiotakos D.B., Yannakoulia M. (2021). Motives for weight loss and weight loss maintenance: Results from the MedWeight study. J. Hum. Nutr. Diet..

[98] Brockmeyer T., Simon J.J., Becker A., Friederich H.-C. (2017). Reward-related decision making and long-term weight loss maintenance. Physiol. Behav..

[99] Levinge E., Stapleton P., Sabot D. (2020). Delineating the psychological and behavioural factors of successful weight loss maintenance. Heliyon.

[100] Gilmartin J., Murphy M. (2015). The effects of contemporary behavioural weight loss maintenance interventions for long term weight loss: A systematic review. J. Res. Nurs..

[101] Cleo G., Beller E., Glasziou P., Isenring E., Thomas R. (2020). Efficacy of habit-based weight loss interventions: A systematic review and meta-analysis. J. Behav. Med..

[102] Hartmann-Boyce J., Boylan A.-M., Jebb S.A., Fletcher B., Aveyard P. (2017). Cognitive and behavioural strategies for self-directed weight loss: Systematic review of qualitative studies. Obes. Rev..

[103] Puhl R.M., Quinn D.M., Weisz B.M., Suh Y.J. (2017). The Role of Stigma in Weight Loss Maintenance Among U.S. Adults. Ann. Behav. Med..

[104] Täuber S., Gausel N., Flint S.W. (2018). Weight Bias Internalization: The Maladaptive Effects of Moral Condemnation on Intrinsic Motivation. Front. Psychol..

[105] Wieland M.L., Njeru J.W., Okamoto J.M., Novotny P.J., Breen-Lyles M.K., Goodson M., Capetillo G.D.P., Molina L.E., Sia I.G. (2020). Association of social network factors with weight status and weight loss intentions among hispanic adults. J. Behav. Med..

[106] Sturgiss E.A., O’bRien K., Elmitt N., Agostino J., Ardouin S., Douglas K., Clark A.M. (2021). Obesity management in primary care: Systematic review exploring the influence of therapeutic alliance. Fam. Pract..

[107] Burgess E., Hassmén P., Pumpa K.L. (2017). Determinants of adherence to lifestyle intervention in adults with obesity: A systematic review. Clin. Obes..

[108] Hutchesson M.J., Rollo M.E., Krukowski R., Ells L., Harvey J., Morgan P.J., Callister R., Plotnikoff R., Collins C.E. (2015). eHealth interventions for the prevention and treatment of overweight and obesity in adults: A systematic review with meta-analysis. Obes. Rev..

[109] Sorgente A., Pietrabissa G., Manzoni G.M., Re F., Simpson S., Perona S., Rossi A., Cattivelli R., Innamorati M., Jackson J.B. (2017). Web-Based Interventions for Weight Loss or Weight Loss Maintenance in Overweight and Obese People: A Systematic Review of Systematic Reviews. J. Med. Internet Res..

[110] Gibson A., Sainsbury A. (2017). Strategies to Improve Adherence to Dietary Weight Loss Interventions in Research and Real-World Settings. Behav. Sci..

[111] Lee P.C., Dixon J.B. (2017). Food for Thought: Reward Mechanisms and Hedonic Overeating in Obesity. Curr. Obes. Rep..

[112] Schoenfeld B.J., Aragon A.A., Krieger J.W. (2015). Effects of meal frequency on weight loss and body composition: A meta-analysis. Nutr. Rev..

[113] Deslippe A.L., Soanes A., Bouchaud C.C., Beckenstein H., Slim M., Plourde H., Cohen T.R. (2023). Barriers and facilitators to diet, physical activity and lifestyle behavior intervention adherence: A qualitative systematic review of the literature. Int. J. Behav. Nutr. Phys. Act..

[114] Horne J., Gilliland J., O’Connor C., Seabrook J., Madill J. (2020). Enhanced long-term dietary change and adherence in a nutrigenomics-guided lifestyle intervention compared to a population-based (GLB/DPP) lifestyle intervention for weight management: Results from the NOW randomised controlled trial. BMJ Nutr. Prev. Health.

[115] Cooper C.B., Neufeld E.V., Dolezal B.A., Martin J.L. (2018). Sleep deprivation and obesity in adults: A brief narrative review. BMJ Open Sport Exerc. Med..

[116] Hirshkowitz M., Whiton K., Albert S.M., Alessi C., Bruni O., DonCarlos L., Hazen N., Herman J., Katz E.S., Kheirandish-Gozal L. (2015). National Sleep Foundation’s sleep time duration recommendations: Methodology and results summary. Sleep Health.

[117] Rogers E.M., Banks N.F., Jenkins N.D.M. (2024). The effects of sleep disruption on metabolism, hunger, and satiety, and the influence of psychosocial stress and exercise: A narrative review. Diabetes Metab. Res. Rev..

[118] Zerón-Rugerio M.F., Doblas-Faxeda S., Diez-Hernández M., Izquierdo-Pulido M. (2023). Are Emotional Eating and Other Eating Behaviors the Missing Link in the Relationship Between Inadequate Sleep and Obesity? A Systematic Review. Nutrients.

[119] Tasali E., Wroblewski K., Kahn E., Kilkus J., Schoeller D.A. (2022). Effect of Sleep Extension on Objectively Assessed Energy Intake Among Adults with Overweight in Real-life Settings: A Randomized Clinical Trial. JAMA Intern. Med..

[120] Kline C.E., Chasens E.R., Bizhanova Z., Sereika S.M., Buysse D.J., Imes C.C., Kariuki J.K., Mendez D.D., Cajita M.I., Rathbun S.L. (2021). The association between sleep health and weight change during a 12-month behavioral weight loss intervention. Int. J. Obes..

[121] Larsen S.C., Horgan G., Mikkelsen M.-L.K., Palmeira A.L., Scott S., Duarte C., Santos I., Encantado J., O’DRiscoll R., Turicchi J. (2020). Consistent sleep onset and maintenance of body weight after weight loss: An analysis of data from the NoHoW trial. PLoS Med..

[122] de Melo C.M., Quaresma M.V.L.d.S., del Re M.P., Ribeiro S.M.L., Antunes H.K.M., Togeiro S.M., Tufik S., de Mello M.T. (2021). One-month of a low-energy diet, with no additional effect of high-protein, reduces Obstructive Sleep Apnea severity and improve metabolic parameters in obese males. Clin. Nutr. ESPEN.

[123] Hudson J.L., Zhou J., Campbell W.W. (2020). Adults Who Are Overweight or Obese and Consuming an Energy-Restricted Healthy US-Style Eating Pattern at Either the Recommended or a Higher Protein Quantity Perceive a Shift from «Poor» to «Good» Sleep: A Randomized Controlled Trial. J. Nutr..

[124] Swift D.L., McGee J.E., Earnest C.P., Carlisle E., Nygard M., Johannsen N.M. (2018). The Effects of Exercise and Physical Activity on Weight Loss and Maintenance. Prog. Cardiovasc. Dis..

[125] Creasy S.A., Hibbing P.R., Cotton E., Lyden K., Ostendorf D.M., Willis E.A., Pan Z., Melanson E.L., Catenacci V.A. (2021). Temporal patterns of physical activity in successful weight loss maintainers. Int. J. Obes..

[126] Zhang H., Tong T.K., Kong Z., Shi Q., Liu Y., Nie J. (2021). Exercise training-induced visceral fat loss in obese women: The role of training intensity and modality. Scand. J. Med. Sci. Sports.

[127] Wewege M., Van Den Berg R., Ward R.E., Keech A. (2017). The effects of high-intensity interval training vs. moderate-intensity continuous training on body composition in overweight and obese adults: A systematic review and meta-analysis. Obes. Rev..

[128] Sardeli A.V., Komatsu T.R., Mori M.A., Gáspari A.F., Chacon-Mikahil M.P.T. (2018). Resistance Training Prevents Muscle Loss Induced by Caloric Restriction in Obese Elderly Individuals: A Systematic Review and Meta-Analysis. Nutrients.

[129] Eglseer D., Traxler M., Schoufour J.D., Weijs P.J.M., Voortman T., Boirie Y., Cruz-Jentoft A.J., Reiter L., Bauer S., SO-NUTS Consortium (2023). Nutritional and exercise interventions in individuals with sarcopenic obesity around retirement age: A systematic review and meta-analysis. Nutr. Rev..

[130] Warburton D.E.R., Bredin S.S.D. (2017). Health benefits of physical activity: A systematic review of current systematic reviews. Curr. Opin. Cardiol..

[131] Tremblay A., Dutheil F., Drapeau V., Metz L., Lesourd B., Chapier R., Pereira B., Verney J., Baker J.S., Vinet A. (2019). Long-term effects of high-intensity resistance and endurance exercise on plasma leptin and ghrelin in overweight individuals: The RESOLVE Study. Appl. Physiol. Nutr. Metab..

[132] Paixão C., Dias C.M., Jorge R., Carraça E.V., Yannakoulia M., de Zwaan M., Soini S., Hill J.O., Teixeira P.J., Santos I. (2020). Successful weight loss maintenance: A systematic review of weight control registries. Obes. Rev..

[133] Spreckley M., Seidell J., Halberstadt J. (2021). Perspectives into the experience of successful, substantial long-term weight-loss maintenance: A systematic review. Int. J. Qual. Stud. Health Well-Being.

[134] Young M.D., Callister R., Collins C.E., Plotnikoff R.C., Aguiar E.J., Morgan P.J. (2017). Efficacy of a gender-tailored intervention to prevent weight regain in men over 3 years: A weight loss maintenance RCT. Obesity.

[135] Hasan B., Nayfeh T., Alzuabi M., Wang Z., Kuchkuntla A.R., Prokop L.J., Newman C.B., Murad M.H., Rajjo T.I. (2020). Weight loss and serum lipids in overweight and obese adults: A systematic review and meta-analysis. J. Clin. Endocrinol. Metab..

[136] Willoughby D., Hewlings S., Kalman D. (2018). Body Composition Changes in Weight Loss: Strategies and Supplementation for Maintaining Lean Body Mass, a Brief Review. Nutrients.

[137] Devries M.C., Sithamparapillai A., Brimble K.S., Banfield L., Morton R.W., Phillips S.M. (2018). Changes in Kidney Function Do Not Differ Between Healthy Adults Consuming Higher-Compared with Lower- or Normal-Protein Diets: A Systematic Review and Meta-Analysis. J. Nutr..

[138] Cheng Y., Zheng G., Song Z., Zhang G., Rao X., Zeng T. (2024). Association between dietary protein intake and risk of chronic kidney disease: A systematic review and meta-analysis. Front. Nutr..

[139] Monnier L., Schlienger J.-L., Colette C., Bonnet F. (2021). The obesity treatment dilemma: Why dieting is both the answer and the problem? A mechanistic overview. Diabetes Metab..

[140] Cheng Z., Zhang L., Yang L., Chu H. (2022). The critical role of gut microbiota in obesity. Front. Endocrinol..

[141] Strain T., Flaxman S., Guthold R., Semenova E., Cowan M., Riley L.M., Bull F.C., Stevens G.A., Raheem R.A., Agoudavi K. (2024). National, regional, and global trends in insufficient physical activity among adults from 2000 to 2022: A pooled analysis of 507 population-based surveys with 5·7 million participants. Lancet Glob. Health.

[142] Paluch A.E., Bajpai S., Bassett D.R., Carnethon M.R., Ekelund U., Evenson K.R., Galuska D.A., Jefferis B.J., Kraus W.E., Lee I.-M. (2022). Daily steps and all-cause mortality: A meta-analysis of 15 international cohorts. Lancet Public Health.

[143] Rodríguez-Gutiérrez E., Torres-Costoso A., Cruz B.d.P., de Arenas-Arroyo S.N., Pascual-Morena C., Bizzozero-Peroni B., Martínez-Vizcaíno V. (2024). Daily steps and all-cause mortality: An umbrella review and meta-analysis. Prev. Med..

[144] Timkova V., Minarikova D., Fabryova L., Buckova J., Minarik P., Katreniakova Z., Nagyova I. (2024). Facilitators and barriers to behavior change in overweight and obesity management using the COM-B model. Front. Psychol..

[145] Pineda E., Stockton J., Scholes S., Lassale C., Mindell J.S. (2024). Food environment and obesity: A systematic review and meta-analysis. BMJ Nutr. Prev. Health.

